# Dynamic Courtship Signals and Mate Preferences in *Sepia plangon*

**DOI:** 10.3389/fphys.2020.00845

**Published:** 2020-08-07

**Authors:** Alejandra López Galán, Wen-Sung Chung, N. Justin Marshall

**Affiliations:** Sensory Neurobiology Group, Queensland Brain Institute, The University of Queensland, St Lucia, QLD, Australia

**Keywords:** cephalopods, reproductive behavior, female choice, ethogram, male competition, body pattern

## Abstract

Communication in cuttlefish includes rapid changes in skin coloration and texture, body posture and movements, and potentially polarized signals. The dynamic displays are fundamental for mate choice and agonistic behavior. We analyzed the reproductive behavior of the mourning cuttlefish *Sepia plangon* in the laboratory. Mate preference was analyzed via choice assays (*n* = 33) under three sex ratios, 1 male (M): 1 female (F), 2M:1F, and 1M:2F. We evaluated the effect of modifying polarized light from the arms stripes and ambient light with polarized and unpolarized barriers between the cuttlefish. Additionally, to assess whether a particular trait was a determinant for mating, we used 3D printed cuttlefish dummies. The dummies had different sets of visual signals: two sizes (60 or 90 mm mantle length), raised or dropped arms, high or low contrast body coloration, and polarized or unpolarized filters to simulate the arms stripes. Frequency and duration (s) of courtship displays, mating, and agonistic behaviors were analyzed with GLM and ANOVAs. The behaviors, body patterns, and their components were integrated into an ethogram to describe the reproductive behavior of *S. plangon*. We identified 18 body patterns, 57 body patterns components, and three reproductive behaviors (mating, courtship, and mate guarding). Only sex ratio had a significant effect on courtship frequency, and the male courtship success rate was 80%. Five small (ML < 80 mm) males showed the dual-lateral display to access mates while avoiding fights with large males; this behavior is characteristic of male “sneaker” cuttlefish. Winner males showed up to 17 body patterns and 33 components, whereas loser males only showed 12 patterns and 24 components. We identified 32 combinations of body patterns and components that tended to occur in a specific order and were relevant for mating success in males. Cuttlefish were visually aware of the 3D-printed dummies; however, they did not start mating or agonistic behavior toward the dummies. Our findings suggest that in *S. plangon*, the dynamic courtship displays with specific sequences of visual signals, and the sex ratio are critical for mate choice and mating success.

## 1. Introduction

Animal communication is a complex mechanism to transfer information between signalers and perceivers (Scott-Phillips, 2008). Communication involves a signaller using specialized morphology or behaviors to influence the current or future behaviors of another individual (Owren et al., 2010). Animals communicate in response to different tasks, including alarm calls, allocation of food, courtship, and mating (Searcy and Nowicki, 2010). Courtship signaling is essential for mate recognition and is frequently multimodal. Animals can use chemical signals (pheromones), vocalizations, color patterns, and movements during courtship displays (Mendelson and Shaw, 2012; Higham and Hebets, 2013). Courtship allows females and males to ensure that they are mating with an animal of the same species, and present information about their quality as a potential mate (Breed and Moore, 2012). There is a growing interest in studying mating signals with bright-colored patterns and intricate courtship displays in both terrestrial and aquatic organisms, such as those in peacock spiders (Girard et al., 2011, 2018; Taylor and McGraw, 2013), birds of paradise (Scholes, 2008; Scholes et al., 2017; Ligon et al., 2018), and siamese fighting fish (Ma, 1995). Cephalopods are renowned for their dynamic displays for courtship and agonistic competitions for potential mates (Hall and Hanlon, 2002; Naud et al., 2004; Allen et al., 2017; Lin et al., 2017; Lin and Chiao, 2018). The development of these elaborate displays is often driven by intense sexual selection, providing an excellent system to study behavior and sexual selection in mating systems (Andersson, 1994).

Most coastal coleoid cephalopods (e.g., octopus, cuttlefish, and squids) have a short life span of one or two years and die shortly after spawning (Jereb and Roper, 2005, 2010; Jereb et al., 2013; Lu and Chung, 2017). Cephalopods also have the most complicated central nervous system of all invertebrates at both anatomical and functional levels (Boycott, 1961; Nixon and Young, 2003; Shigeno et al., 2018), and possess unique colorblind camouflage, mimicry, and communication abilities (Hanlon and Messenger, 2018). Cephalopod dynamic body patterns are directly controlled by their brain and continuously adapt to match the visual perception of the environment, communicate with mates, and solve different tasks (Boycott, 1961; Darmaillacq et al., 2014; Liu and Chiao, 2017; Gonzalez-Bellido et al., 2018; Hanlon and Messenger, 2018). These dynamic body patterns are composed of multiple chromatic, textural, locomotor, and postural components simultaneously expressed (Packard and Hochberg, 1977). For example, the Intense Zebra pattern of the mature male *Sepia officinalis* has white and black zebra bands on the mantle, white and dark fin spots, dilated pupils (chromatic components), smooth skin (textural components), dropping arms and extended fourth arm (postural components), and hovering display (locomotor components). This pattern is displayed by mature males cuttlefish, and it is used for sex recognition and agonistic behavior (Hanlon and Messenger, 1988).

Coleoid cephalopods have evolved several reproductive strategies in response to sexual selection. For instance, the hectocotylus, ligula, and calamus in males are morphological adaptations to transfer the sperm to the females (Voight, 1991, 2002; Thompson and Voight, 2003). Alternative mating tactics in squids and cuttlefish enhance the mating opportunities of small males by avoiding male competitions (Hanlon et al., 2002; Wada et al., 2005; Zeidberg, 2009; Brown et al., 2012; Lin and Chiao, 2018; Marian et al., 2019). Male octopus mate “at a distance” to escape and avoid sexual cannibalism (Hanlon and Forsythe, 2008; Huffard and Bartick, 2015). Promiscuity, mating aggregations, and sperm competition are also behavioral adaptations related to sexual selection in cephalopods (Hall and Hanlon, 2002; Jantzen and Havenhand, 2003; Naud et al., 2004; Morse and Huffard, 2019). For instance, several investigations have shown that multiple paternity occurs in some species, such as *Octopus minor* (Bo et al., 2016), *Octopus vulgaris* (Quinteiro et al., 2011), and the cuttlefish *Sepiella japonica* (Liu et al., 2019). Other studies in squids have found a sequence of pattern-based signals associated with determining dominance in reproductive battles (Lin et al., 2017; Lin and Chiao, 2018).

Current knowledge of cuttlefish reproductive interactions and visual signals is based primarily on four large-sized species [Mantle length (ML) of mature individuals > 300 mm]: (1) *Sepia apama* (Hall and Hanlon, 2002; Zylinski et al., 2011; Schnell et al., 2015a,b, 2019), (2) *Sepia latimanus* (Roper and Hochberg, 1988; Adamo and Hanlon, 1996; How et al., 2017; Hanlon and Messenger, 2018) (3) *Sepia officinalis* (Hanlon and Messenger, 1988; Adamo and Hanlon, 1996; Boal, 1997; Hanlon et al., 1999; Chiao et al., 2005; Mäthger et al., 2009), and (4) *Sepia pharaonis* (Lee et al., 2016; Nakajima and Ikeda, 2017). Over the past 30 years, additional pattern, textural, postural and locomotor components have been documented in these species. Each has species-specific body patterns, but they also share a high degree of similarity in some courtship body patterns common across many day-active shallow-water cephalopods. For example, the male zebra pattern (white and dark zebra bands across the mantle, arms and/or fins), and the passing cloud display characterized by coordinated waves of expanded chromatophores flowing as dark bands over the body (Hanlon and Messenger, 1988; How et al., 2017). Another iconic cuttlefish species is the small (ML < 110 mm) flamboyant cuttlefish, *Metasepia pfefferi*, which possesses over 100 display components (Roper and Hochberg, 1988; Thomas and MacDonald, 2016). *Metasepia pfefferi* is capable of showing elaborate body patterns, with three pairs of large and flap-like papillae in the dorsal mantle, long papillae over eyes, and passing clouds running in several directions simultaneously (Roper and Hochberg, 1988; Jereb and Roper, 2005; Laan et al., 2014; Thomas and MacDonald, 2016). Although many studies have demonstrated the complexity of cuttlefish visual signaling, the temporal structure of the multiple behavioral displays associated with reproduction is poorly-known. To date, only one study has analyzed the temporal order of the body pattern components expressed by the squid *Sepioteuthis lessoniana* during reproductive interactions (Lin et al., 2017).

Here we selected a relatively small-sized cuttlefish, *Sepia plangon*, which inhabits seagrass beds around Australian coastal waters (living depth < 85 m) (Jereb and Roper, 2005). Brown et al. (2012) reported the mating behavior of this species in the wild and in captivity. Unlike other large cuttlefish species (Dunn, 1999; Hall and Hanlon, 2002; Naud et al., 2004), *S. plangon* apparently does not form large aggregations for mating. As Brown et al. (2012) reported, this species forms small groups on the spawning grounds. Male-only assemblages (32.50%), male-female pairs (1M:1F, 25.00%), and groups of two males with one female (2M:1F, 12.50%) were the most frequent. Interestingly, five small males (ML < 80.00 mm) displayed a deceptive body pattern in the presence of a female and a larger male. This dual-lateral display consisted of two simultaneous body patterns, one on each half of the mantle. Males showed the typical male coloration toward their counterparts but displayed the female appearance to a larger male to avoid fighting (Brown et al., 2012). In addition to the repertoire of visual signals, *S. plangon* spawns multiple times (Beasley et al., 2017), making this species suitable for observation of repeated mating behavior.

**Figure 1:**
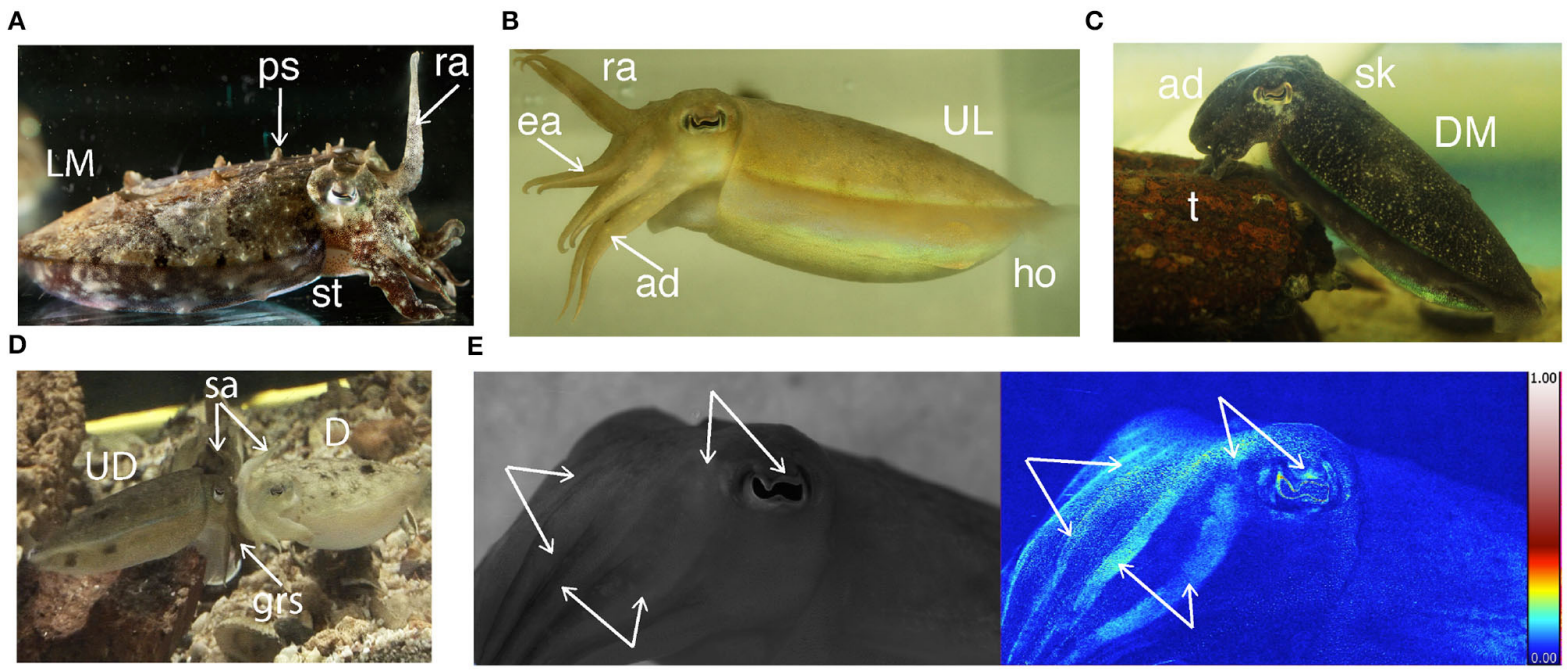
Some of the body patterns of *S. plangon*. **(A)** Male with raised arms (ra), papillate skin (ps), sitting (st), and light mottle (LM). **(B)** Female. Hovering (ho), raised I arms (ra), extended II and III arms (ea), dropped arm (ad), and uniform light (UL) pattern. **(C)** Female in tripod posture (t), dropped arms (ad), smooth skin (sk), and dark mottle pattern. **(D)** Female and male in head-to-head mating position with splayed arms (sa), male grasping (grs), uniform darkening (UD), and deimatic pattern (D). **(E)** Male cuttlefish. The white arrows refer to polarized arm stripes and polarized eye sclera. The blue-red scale bar represents the percentage of polarization from 0 (blue) to 1 (white) through (red) which is typical for polarization in nature.

*S. plangon* has a single type of visual pigment (λmax 499 nm), suggesting that this species, like other cuttlefish species (Marshall and Messenger, 1996), is unable to detect or respond to colors through their retina (Chung and Marshall, 2016). *Sepia plangon* does possess orthogonally-arranged photoreceptors enabling sensitivity to polarized (PL) signals. This species may discriminate 1^0^ e-vector difference, the highest PL resolution in animals with PL vision studied to date (Talbot and Marshall, 2010a,b; Temple et al., 2012). Additionally, *S. plangon* reflects PL light via iridophores located under the skin, forming strong PL signals on arms, the frontal area of the head, and around eyes (Figure 1). These signals are similar to those of *S. officinalis* (Shashar and Cronin, 1996; Shashar et al., 1996, 2002; Boal et al., 2004; Chiou et al., 2007; Mäthger and Hanlon, 2007; Mäthger et al., 2009; Cartron et al., 2013a,b,c). Several studies have proposed that PL vision enables a covert communication channel in cephalopods as many other animals are unable to detect the PL signals (Moody and Parriss, 1961; Jander et al., 1963; Tasaki and Karita, 1966; Saidel et al., 1983, 2005; Shashar and Cronin, 1996; Shashar et al., 1996, 2000, 2002; Boal et al., 2004; Talbot and Marshall, 2010b; Cartron et al., 2013a,b,c; Marshall et al., 2019). However, despite decades of study, the evidence is inconclusive and no behavioral function has been attributed to these signals.

To understand whether ambient light conditions affect mate choice, and which visual signals may influence the outcome of mating competitions, we selected *S. plangon* which can be reared in captivity and tested under different light conditions. First, we investigated the courtship and mate choice under different sex ratios. We evaluated whether the presence of polarized (POL) vs. unpolarized (UNPOL) barriers would affect the reproductive behavior of *S. plangon*, from courtship to mating. For example, we tested whether *S. plangon* started courtship and attempted to mate regardless of a barrier limiting physical contact between them (UNPOL), and regardless of a polarized filter (POL). Then, we used 3D resin-printed cuttlefish dummies with one static component of specific body patterns (e.g., large vs. small body size; PL vs. non-PL arm stripes, uniform light, dark uniform, weak zebra, strong zebra). We aimed to test if each component alone could trigger a response related to mating behavior, to ultimately understand the effect of each separate component in the complex courtship behavior of *S. plangon*.

## 2. Methods

### 2.1. Animal Collection and Care

We collected 34 mature females and 32 males using seine nets in Dunwich, North Stradbroke Island, Queensland, Australia, between April-July 2016, August-October 2017, and Feb-May 2018. Our study was carried out following the permits by The University of Queensland - Animal Ethics Committee (permit number QBI/304/16), Queensland Government - Department of National Parks, Sports and Racing (Moreton Bay Marine Park Permit QS2018/CVL625), and Queensland Government - Department of Primary Industries and Fisheries (General Fisheries Permit 180731). Cuttlefish were placed individually in housing tanks of (610 × 600 × 450 mm) with running seawater, which was monitored continuously using a filtered re-circulating water system (water temperature 20–24^o^C, salinity 35–36 psu, light-dark cycle 12–12 h) at Moreton Bay Research Station (MBRS). All tanks contained sand, rocks, and PVC tubes as substrates, and the cuttlefish were fed daily with live prey (e.g., glass shrimps and purple shore crabs *Hemigrapsus*) *ad libitum*. The animals were kept in housing tanks for at least a week before starting the behavioral experiments.

### 2.2. Pre-copulatory, Mating, and Postcopulatory Behavior

**Figure 2:**
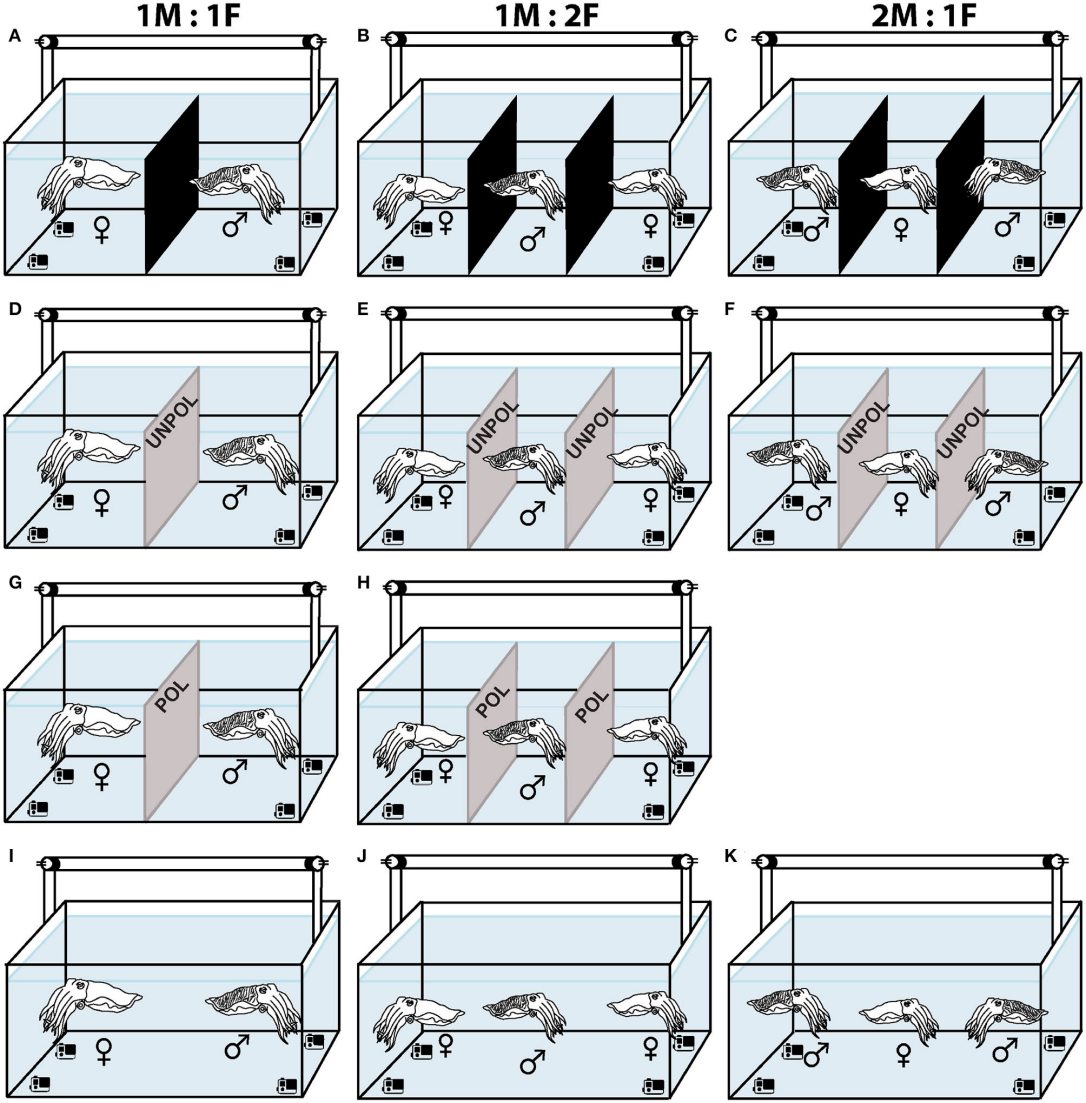
Schematic representation of the experimental design for mate choice. ♀ = Females. ♂ = Males. **(A)** One pair and three cuttlefish **(B,C)** with black dividers to limit visual contact before the start of the experiments. **(D)** Two cuttlefish with an unpolarized (UNPOL) barrier between them. **(E,F)** Three cuttlefish with unpolarized barrier in the tank. **(G)** A male and a female with a polarized (POL) barrier between them. **(H)** Three cuttlefish with polarized barrier in the tank. **(I–K)** Two or three cuttlefish in control (DIRECT) condition. **(A,D,G,I)** Sex ratio = 1M:1F. **(B,E,H,J)** = 1M:2F. **(C,F,K)** = 2M:1F.

To analyze the effect of sex ratio in courtship behavior, a pair (a female and a male 1M:1F), or three cuttlefish (two males and a female 2M:1F, or one male and two females 1M:2F) were placed in a tank with a black acrylic divider to limit the visual contact for an hour prior to the start of each experiment (Figures 2A–C). Next, we removed the dividers between the tanks and recorded the cuttlefish interactions with four underwater cameras for at least an hour, or until animals stop interacting (1.5 h max) (Figures 2I–K).

Then using BORIS 7.7.4® Friard and Gamba (2016) to analyse the videos, we created a catalog of reproductive behaviors, agonistic fights, courtship, mating, mate guarding, and body patterns of *S. plangon*. The textural (skin texture), chromatic (body color), postural (body position), locomotor (body movement), and polarization components of the body patterns of *S. plangon* were also identified. We followed the body pattern descriptions by Hanlon and Messenger (1988), Borrelli et al. (2005), Schnell et al. (2015b), Thomas and MacDonald (2016), How et al. (2017) and Nakajima and Ikeda (2017) (Table 1). The body patterns were categorized into two groups: (1) Acute pattern (body patterns displayed for less than 5 min); (2) Chronic patterns (those lasting over 5 min) (Table 1). Furthermore, we classified the components of these body patterns in two categories: (1) Point events with a duration of 5 s or less, and (2) State events, which were visible for more than 5 s (Table 1).

**Table 1:** Body patterns and their components of *S. plangon* and the alphabetic code used in this study.

**Components - Body patterns**	**Code**				
**REPRODUCTIVE BEHAVIORS (3)**					
Courtship display Ω	C	Mate Guarding Ω	MG	Mating Δ	M
**BODY PATTERNS (18)**					
Chromatic Pulse Π	CP	Dark Mottle Π	DM	Deimatic Π	D
Dual-Lateral Display Π	DLD	Dynamic Polarization SignalsΠ	DPS	Flamboyant Π	F
Intense Zebra Π	IZ	Lateral Display Π	LD	Light Mottle Ψ	LM
Multidirectional Passing Wave Display Π	MDPWD	Shovel Display Π	SHD	Strong Disruptive Π	STD
Stipple Π	ST	Uniform Blanching Π	UB	Uniform Darkening Π	UD
Uniform Light Ψ	UL	Weak Disruptive Ψ	WD	Weak Zebra Ψ	WZ
**CHROMATIC COMPONENTS (28)**					
Anterior Head Bar Δ	ahb	Anterior Mantle Bar Δ	amb	Anterior Transverse Mantle Line Δ	atml
Dark Arms Δ	da	Dark Arm Stripes Δ	das	Dark Eye Ring Δ	der
Dark Fin Spots Δ	dfs	Dark Zebra Bands Δ	dzb	Dilated Pupil Δ	dp
Large White Mantle Spots Δ	lwms	Latero-Ventral Patches Δ	lvp	Mantle Margin Scalloping Δ	mmsc
Mantle Margin Stripe Δ	mmst	Median Mantle Stripe Δ	mms	Paired of Mantle Spots Δ	pms
Posterior Head Bar Δ	phb	Posterior Mantle Bar Δ	pmb	Posterior Transverse Mantle Line Δ	ptml
White Arm Spots Δ	was	White Fin Spots Δ	was	White Head Bar Δ	whb
White Major Lateral Papillae Δ	whb	White Mantle Band Δ	wmb	White Neck Spots Δ	wns
White Posterior Triangle Δ	wpt	White Splotches Δ	ws	White Square Δ	wsq
White Zebra Bands Δ	wzb				
**POSTURAL COMPONENTS (14)**					
Arms Dropped Δ	ad	Bipod Δ	bi	Buried Δ	b
Elongated body Δ	eb	Extended II, III Arms Δ	ea	Extended IV Arms Δ	eaf
Flanged Fin Δ	ff	Flattened Body Δ	fb	Fully Extended I - IV Arms Δ	fe
Raised I, II Arms Δ	ra	Raised Head Δ	rh	Sitting Ω	st
Splayed Arms Δ	sa	Tripod Ω	t		
**LOCOMOTOR COMPONENTS (12)**					
Ambling Ω	a	Flee Δ	f	Forward Rush Δ	fr
Grappling Ω	grp	Grasping Ω	grs	Hiding Ω	h
Hovering Ω	ho	Inking Δ	i	Swimming Ω	sw
Turning toward a Mate Δ	ttm	Water Jetting Δ	wj	Waving arms Ω	wa
**IRIDISCENT COMPONENTS (6)**					
Iridescent Eye Sclera Δ	is	Iridescent Green/Blue Arm Stripes Δ	igas	Iridescent Green/Blue Mantle Margin Stripe Δ	igmm
Iridescent Pink/Orange Arm Stripes Δ	ipas	Iridescent Pink/Orange Mantle Margin Stripe Δ	ipmm	Iridescent Ventral Mantle Δ	ivm
**TEXTURAL COMPONENTS (3)**					
Coarse Skin Δ	cs	Papillate Skin Δ	ps	Smooth Skin Ω	sk

*State component = Ω, Point component = Δ, Acute pattern = Ψ, Chronic pattern = Π*.

### 2.3. Polarized vs. Non-polarized Barriers

Next, to study the effect of light conditions on the reproductive behavior of *S. plangon*, we put polarized (POL) or unpolarized (UNPOL) neutral density barriers between the cuttlefish and recorded the behavioral interactions in the three sex ratios mentioned above (1M:1F, 2M:1F, and 1M:2F, Figures 2D–H). Due to the small number of males collected for our study, 2M:1F - POL trials were not conducted, (see Supplementary Material for more details). The polariser filter was a 42.00% Transmission Neutral Gray Acrylic Laminated Linear Polarizer (AP27-024T, American Polarizers Inc., USA). The filter was horizontally aligned and attached to a frame made of PVC tubes. A sheet of a white diffuser (PTFE sheet, Dotmar EPP Pty Ltd, Australia) was attached to the light source (Arlec 2x20W LED Work light, Arlec Australia Pty Ltd) placed above the tank. A piece of 0.3 soft neutral density filters (Lee Filters, UK) were glued to a float glass window on both sides to make the unpolarized barrier. Using the same video analysis method, we identified the chromatic, textural, postural, and locomotor components of the body patterns that *S. plangon* used for reproductive behavior under different light conditions. POL filters modified the polarization signals from arm stripes and body of *S. plangon*; therefore, a cuttlefish could barely see the polarized signals from a mate placed at the other side of the filter.

### 2.4. Statistical Analysis

We analyzed the frequency and duration (in seconds, sec) of the courtship, mating, agonistic fights, and mate guarding. Due to a large number of zeros from our frequency data (animals that did not start courtship or mate), we used generalized linear models (GLM) with negative binomial (NB) distribution. The duration was analyzed with two-way factorial ANOVAs, using sex ratio as one factor of 3 levels—1M:1F, 1M:2F, 2M:1F, and type of barrier as the second factor of two levels—Polarized (POL) or Unpolarized (UNPOL). Cuttlefish like other cephalopods use their body patterns for communication; therefore, to demonstrate that a specific sequence of signals is determinant for mate choice in *S. plangon*, we transformed each body pattern and component to alphabetic codes of one to five letters (Table 1). Consecutively, we analyzed the data using text mining methods (Silge and Robinson, 2016) to estimate the frequency and the association of body patterns, such as radar charts and correlations with Bonferroni correction for multiple comparisons. All the analyses were conducted in (RStudio Team, 2015) v1.2.1335® (RStudio Team, 2015) using the packages FactoMineR v1.42 (Lê et al., 2008), tidyverse v1.2.1 (Wickham et al., 2019a), tidytext v0.2.2 (Silge and Robinson, 2016), dplyr v0.8.3 (Wickham et al., 2019b), widyr v0.1.2 (Robinson, 2019), tokenizers v0.2.1 (Mullen et al., 2018), quanteda v1.5.1 (Benoit et al., 2018) and igraph v1.2.4.1 (Csardi and Nepusz, 2006).

### 2.5. 3D Printed Cuttlefish Models

We selected five body pattern components from those observed in successful mating (see the details in results, and Table 1) for further tests using 3D printed resin cuttlefish. We downloaded the 3D models from CGTrader (https://www.cgtrader.com, see Appendix for more details). Then, we edited them using the software Blender® version 2.79. The models were printed using Stereolithography to 0.1 mm layer thickness, using a Form2 (Formolabs®) 3D printer at the Australian National Fabrication Facility, Queensland Node (ANFF-Q), and The University of Queensland Library 3D-printing facilities. Dummies (DUM) of two sizes (60 or 90 mm ML) were compared to analyze the effect of body size in mating choice. We measured the importance of body posture using dummies with arms extended or dropped. The models were painted with acrylic paints to simulate four body patterns, such as uniform light (UL) and dark mottle (DM) for females, or intense zebra (IZ) and weak zebra (WZ) for males. We attached stripes of polaroid or neutral-density filter to the arms of the dummies to simulate polarized and unpolarized arm stripes, respectively. All dummies were attached to a thin fishing line to place them into the testing tank. For this experiment, we counted a successful mate choice if a cuttlefish showed interest in the dummy, either by initiating courtship or attempting to mate. Negative results were assigned if cuttlefish ignored, showed aggressive behavior, or remained distant from the dummies. We considered agonistic behavior as a negative result because we used the dummies to test whether the static body pattern could trigger courtship and mating behavior, as all these trials were intersexual experiments (male dummy for females, and female dummies for males).

## 3. Results

We collected 34 mature females and 32 males. Females were larger than males, with mantle length (ML) (mean ± SD) = 74.92 ± 13.02 mm, and total length (TL) = 103.55 ± 21.23 mm. Males ML was 65.62 ± 10.15 mm, and TL = 90.41 ± 13.66 mm. We analyzed the behavior of *S. plangon* during 41h of video analysis. The data were collected from 17 control experiments (no barrier between cuttlefish), four POL trials, four UNPOL observations, and eight DUM experiments (*n* = 33). Nine males were allocated to 1M:1F trials, nine more to 1M:2F condition, and 14 males in 2M:1F tests. Twenty-one males initiated courtship displays (65.63% from the total), and 17 males mated at least once, representing 80.95% success rate of courtship displays. On the other hand, nine females were tested in 1M:1F trials, 18 females in 1M:2F, and seven more in 2M:1F observations, but only 16 females mated (47.06% from the total).

### 3.1. Body Patterns and Courtship Display of *Sepia plangon*

**Figure 3:**
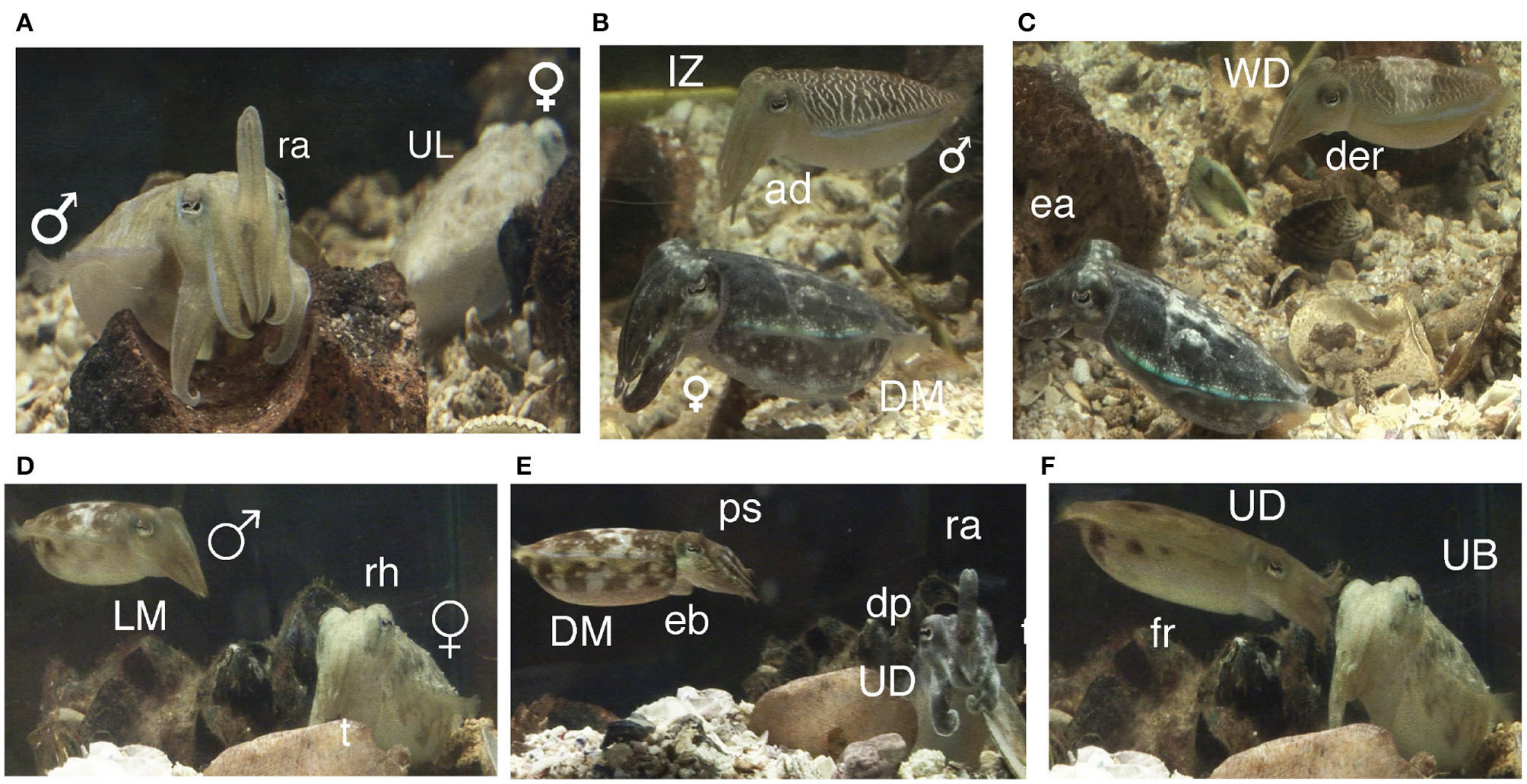
Signals and body patterns of *Sepia plangon* during courtship. The images were from the same pair during the same interaction. **(A)** Before courtship, a male with raised arms (ra) and female with uniform light pattern (UL). **(B)** Male began the courtship with Intense Zebra pattern (IZ) and arms dropped (ad), whereas female showed Dark Mottle pattern (DM). **(C)** Male transitioning to Weak Disruptive (WD) and dark eye ring (der). Female maintained rejection signal (Dark mottle) and extended arms (ea). **(D)** Male with light mottle (LM). Female in tripod posture (t) and raised head (rh). **(E)** Male with elongated Body (eb), DM, and papillate skin (ps). Female showed Uniform Darkening (UD), dilated pupils (dp) and ra. **(F)** Female switched to uniform blanching pattern (UB) while male with UD coloration rushed forwards (fr) to touch the female's head.

We identified a total of 18 body patterns in *S. plangon* (Table 1, Figures 1, 3). Males exhibited all 18 patterns, whereas females only showed 12. Following the general classification of cephalopods' body patterns, we classified them into two categories, Acute and Chronic (Table 1).

#### 3.1.1. Acute Body Patterns

Eleven body patterns with a brief duration fell under this category. Acute patterns had a duration from seconds (sec) to a few minutes.

− 1. Chromatic pulse (CP, mean duration ± standard deviation = 50.75 ± 34.36 sec) (How et al., 2017), also known as “passing clouds” (Hanlon and Messenger, 1988), was a dynamic expansion and contraction of chromatophores to produce bands running in a single direction across the body.
− 2. Deimatic pattern (D, 28.48 ± 25.89 sec) (Hanlon and Messenger, 1988) was characterized by paling and flattening of the body, a pair mantle spots (pms), dark eye rings (der), dilated pupils (dp), and smooth skin (SK).
− 3. Dual-lateral display (DLD, 141.35 ± 252.70 sec) (Brown et al., 2012) was characteristic of small males during agonistic contests. This pattern incorporated two patterns simultaneously. Males mimicked female coloration by showing light or dark mottle pattern in one half of the mantle. This strategy was used to avoid fighting with rivals. However, in the other half of the mantle, “sneaker males” showed the typical male coloration (intense or weak zebra) to the female.
− 4. Dynamic polarization signals (DPS, 191.10 ± 353.20 sec) (Supplementary Video 3): this hitherto undescribed pattern involved dynamic expansion and contraction of chromatophores only in areas where cuttlefish reflects polarized light (e.g., around the eyes, and in the arm stripes), producing bands running in a single direction in these regions. This pattern was expressed exclusively during courtship by males.
− 5. Flamboyant (F, 43.69 ± 43.68 sec) (Hanlon and Messenger, 1988) included papillate skin (ps), splayed arms (sa), dark mottle (DM) coloration, and latero-ventral patches (lvp). *Sepia plangon* displayed flamboyant primarily in the context of defense, but the males also showed this pattern if the females rejected any mating attempt by dropping the arms or moving away from the male.
− 6. Intense zebra (IZ,930.14 ± 1144.17 sec) (Hanlon and Messenger, 1988) was exclusive to males, and it included dark and white zebra bands (dzb, wzb) on the mantle, dark eye rings, smooth skin, and extended IV arms toward another male (eaf).
− 7. Lateral display (LD, 36.26 ± 49.15 sec) (Schnell et al., 2015a) was an agonistic signal from males characterized by light and dark moving bands over the mantle (chromatic pulse in the present study), with the body-oriented laterally to rivals and dark arms or face.
− 8. Multidirectional passing wave display (MDPWD, 214.86 ± 221.81 sec) (How et al., 2017) was similar to chromatic pulse; however, the bands moved in different directions across the body. We observed that small males exhibited this coloration when the females rejected them during courtship.
− 9. Shovel Display (SHD, 190.38 ± 290.26 sec) (Schnell et al., 2015a) incorporated the mantle raised, and rigid arms extended in a shovel-like shape. Large male *S. plangon* produced this pattern as an aggressive signal at the beginning of every male contest (See Supplementary Figure 1).
− 10. Uniform Blanching (UB, 59.16 ± 25.39 sec) (Hanlon and Messenger, 1988) was characterized by a fast retraction of all chromatophores creating a pale appearance.
− 11. Uniform Darkening (UD, 83.57 ± 20.89 sec) (Hanlon and Messenger, 1988) was a quick expansion of all chromatophores seen as an instant darkening of the body.

#### 3.1.2. Chronic Body Patterns

The duration of these displays extends to several minutes. This category encompassed seven body patterns.

− 1. Strong Disruptive (STD, 231.00 ± 323.33 sec) (Hanlon and Messenger, 1988) comprised bold transverse and longitudinal components, both light and dark.
− 2. Weak Disruptive (WD, 578.76 ± 673.95 sec) (Hanlon and Messenger, 1988) is similar to strong Disruptive, but the contrast between dark and light components is less vivid. Both STD and WD had a maximum duration of 846.49 sec.
− 3. Dark Mottle (DM, 953.54 ± 1068.44 sec) (Hanlon and Messenger, 1988) had white and dark dots distributed in the arms, head, and dorsal mantle.
− 4. Light Mottle (LM, 781.52 ± 765.29 sec) (Hanlon and Messenger, 1988) made the overall body tone pale with some of the dark chromatophores expressed as spots or splotches.
− 5. Uniform Light (UL, 263.18 ± 326.51 sec) (Hanlon and Messenger, 1988) had a body coloration similar to white, as the expansion of chromatophores was minimum.
− 6. Stipple (ST, 64.71 ± 61.15 sec) (Hanlon and Messenger, 1988) included light body coloration with small dark spots due to the partial expansion of some chromatophores and papillate skin.
− 7. Weak Zebra (WZ, 673.51 ± 710.70 sec) (Hanlon and Messenger, 1988) was a low-contrast zebra patterning, with white and black zebra bands covering the mantle. Males showed this body color throughout courtship displays.

### 3.2. Courtship, Agonistic, and Mating Behavior

**Figure 4:**
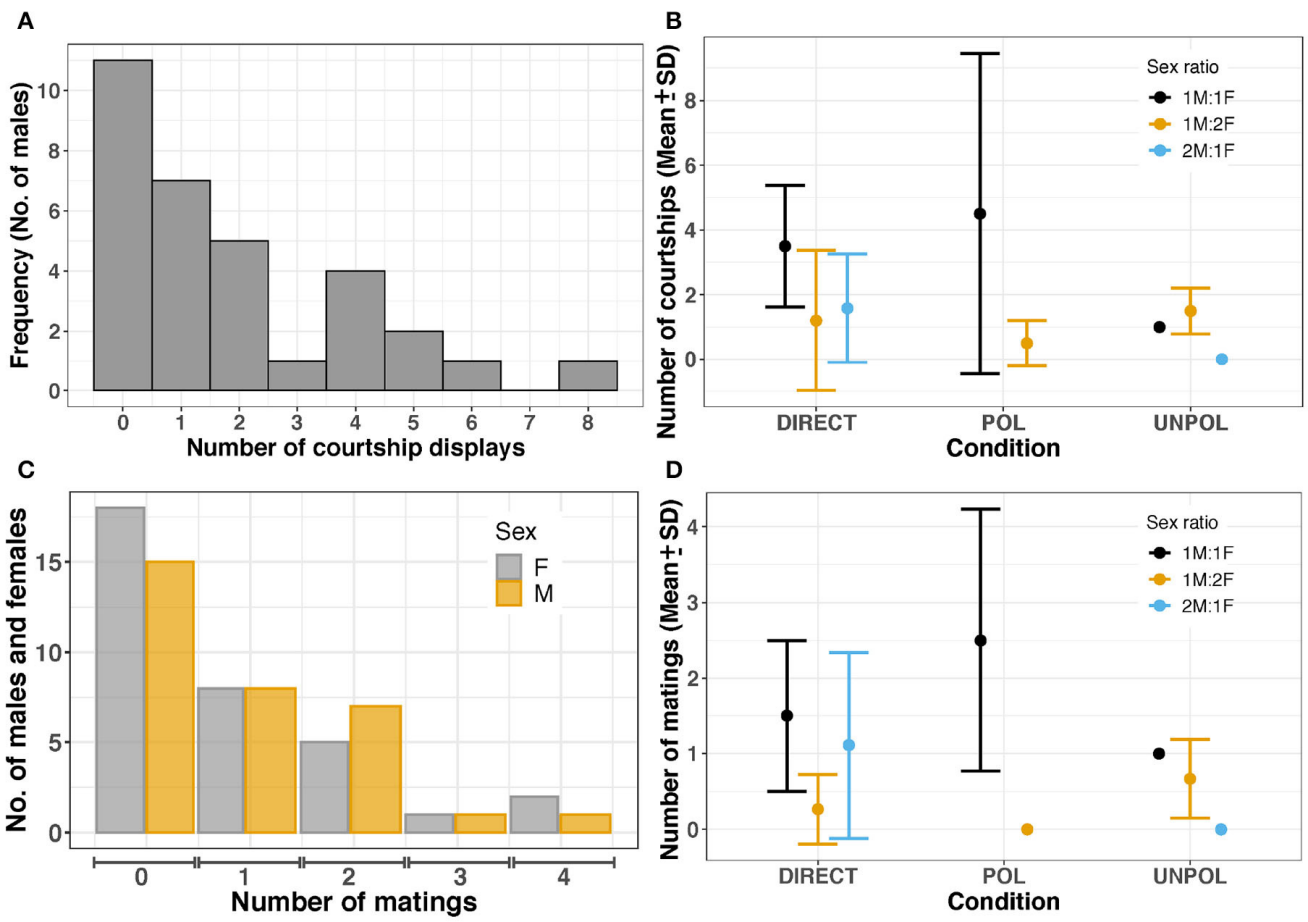
**(A)** Total frequency of courtship displays by males **(B)** Frequency of courtship displays. Large dots and bars represent the mean and standard deviation, respectively. DIRECT, control (no barrier); POL, polarized barrier; UNPOL, unpolarized barrier. Sex ratio = 1M:1F in black, 1M:2F in yellow, 2M:1F in blue. **(C)** Mating frequency in females and males. **(D)** Mean and standard deviation of mating frequency in both males and females. No courtships were observed in 2M:1F - UNPOL experiments. In 1M:2F - POL trials (*n* = 2), only one male started courtship. No matings were observed in 1M:2F - POL and 2M:1F - UNPOL trials.

Before courtship, cuttlefish often camouflaged with light mottle, weak disruptive, or stipple patterns to match with the background of the testing tank. After 1–32 min, males turned toward one female and initiated a courtship display (C) in 20 trials (60.61%). Mating was observed at least once in 17 of 33 experiments (courtship success = 85.00%). *Sepia plangon* had multiple matings (Figure 4), and all the males that mated showed at least one courtship display to the female. Mating was not observed without prior courtship display.

The courtship started when a male showed quick changes of body patterns to the female, and it lasted until both cuttlefish adopted the mating position (head-to-head). Courtship excluded agonistic encounters between males and mating. Courtship latency was then the time before males showed any courtship behavior, and it had a duration of 60.00 to 1923.50 sec. Males initiated courtship displays with the repetitive sequence of seven fast changes of body patterns (Figure 3). The courtship display was formed with an orderly sequence of body patterns starting with light mottle, followed by intense zebra, weak zebra, dark mottle, uniform blanching, uniform darkening, and dark mottle (Figure 3). Intense zebra was continuously observed in 2M:1F trials, but males in 1M:1F and 1M:2F trials showed intense zebra pattern only at the beginning of courtship display. The components of the body patterns during courtship quickly changed from smooth to papillate skin, elongated body (eb), raised arms (ra), flattened body (fb), fully extended I - IV arms (fe), and forward rush (fr) (Figures 3E,F).

Agonistic signals between males encompassed intense zebra display with dark eye rings, extended IV arms to push competitors away from the female, lateral display, raised head, and shovel display. Escalation to agonistic fights was observed only during one trial, (2M:1F with no barrier between cuttlefish) where two males engaged in three aggressive fights. The ML difference between males that showed only agonistic signals was 13.64 ± 9.33 mm. On the other hand, males that initiated agonistic contests had a size difference in ML of 6.48 mm. The average size of winner males was 64.13 ± 11.67 mm of ML, whereas, loser males had 65.49 ± 9.09 mm of ML.

Similar to other cuttlefish, *S. plangon* mated in head-to-head position (Figure 1D). Eight females (23.53% from all females) and eight males (25.00% from all males) coupled only once, five females (14.71%) and seven males (21.88%) mated twice, one female (2.94%) and a male (3.13%) had three copulations, and two females (5.88%) and a male (3.13%) had four.

#### 3.2.1. Sex Ratio

In 1M:2F experiments, the males first chose a female to court but also approached and courted the other female if rejected by the first mate. Rejection signals by the females consisted of dark mottle coloration, dark eye rings, dropped arms, and moving away from the male.

In 2M:1F observations, the males established dominance using agonistic signals. Agonistic signals between males encompassed intense zebra display with dark eye rings, extended IV arms to push competitors away from the female, shovel, and lateral display. Cuttlefish presented these agonistic signals only in six control experiments (2M:1F), where cuttlefish had no barrier between them. Escalation to physical fights included animals grappling (grp) their opponent and inking (See Supplementary Figure 1). We observed male-male fight only in one trial; hence, male dominance was established primarily by agonistic visual signals. The dominant male remained close to the female and was the first to start the courtship display. Furthermore, the dominant males were the first to mate with the female. Five “sneaker” males (see Supplementary Figure 1) avoided fights by simulating female coloration in five control trails (2M:1F), and this strategy led to successful matings by four males (80.00% success rate). Dominant males guarded the female (Mating Guarding, MG) before, during, and after copulation. MG consisted of males with Intense Zebra coloration, or dark mottle, hovering close to the females while extending the IV arms toward competitors. Small males also guarded the paired female, but only in the absence of another competitor.

#### 3.2.2. Polarized / Unpolarized Barriers

POL and UNPOL barriers did not prevent cuttlefish from attempting to mate (See Supplementary Figure 1), as we observed five females and males attempting to mate while they were separated by the POL (*n* = 2) and UNPOL (*n* = 3) barriers (See Supplementary Figure 1). Similar body patterns and behaviors were observed in these trials to those seen in experiments without a barrier, such as courtship display by males (including forward rush trying to push the barrier), females raising the first pair of arms, and both the female and male adopting the head-to-head mating position with spread arms. Mating duration in POL and UNPOL observations was measured from the moment when both cuttlefish attempted to mate. Cuttlefish spread their arms and moved toward each other to adopt the head-to-head mating position despite of the barrier between them. We considered the end of a mating attempt when both cuttlefish moved apart. We observed males and females pushing the barrier trying to reach each other during the matting attempts.

### 3.3. Frequency of Courtship Displays, Male Competitions, and Copulations

#### 3.3.1. Frequency

*Sepia plangon males* males had an average of 1.88 ± 2.11 courtship displays. The maximum number of courtship displays was eight in a 1:1 - POL experiment. Males courted females in all 1M:1F trials, five of nine 1M:2F experiments, and all 2M:1F trials (Figure 4). Cuttlefish in 1:1 - POL condition showed more courtship displays than cuttlefish in the other conditions tested (4.50 ± 4.95). However, the negative binomial GLM suggested that sex ratio was the only variable with a significant effect on courtship frequency, as it was more likely that any male initiated courtship in 2M:1F condition (*b*=−0.949, *p*=0.026) than 1M:2F (*b*=−1.017, *p*=0.034). Four male cuttlefish started courtship displays in 1M:1F, but they were unable to attract females for successful mating. The courtship frequency of the four loser males was 2.25 ± 1.97, whereas in winner males it was 2.94 ± 1.98.

The most frequent agonistic signal was dark eye ring with a maximum of 73 counts (19.90 ± 19.91), followed by intense zebra (max frequency = 16, 3.78 ± 4.27), extended IV arms (max frequency = 32, 1.16 ± 5.66), raised head (max = 5, 0.56 ± 1.22), shovel display (max = 8, 0.38 ± 1.43), mate guarding (max = 2, 0.16 ± 0.45), and lateral display (max = 2, 0.09 ± 0.39). Furthermore, winner males showed agonistic signals more frequently than losers, particularly dark eye rings and intense zebra pattern.

Overall, the mating frequency was 0.86 ± 1.11, but the highest number of copulation attempts (due to the physical limitation by a barrier) was observed in POL - 1:1 condition (2.50 ± 1.73) (Figure 4D); however, GLM did not find any effect by Sex ratio or POL condition on mating frequency (*p* > 0.05).

#### 3.3.2. Duration

**Figure 5:**
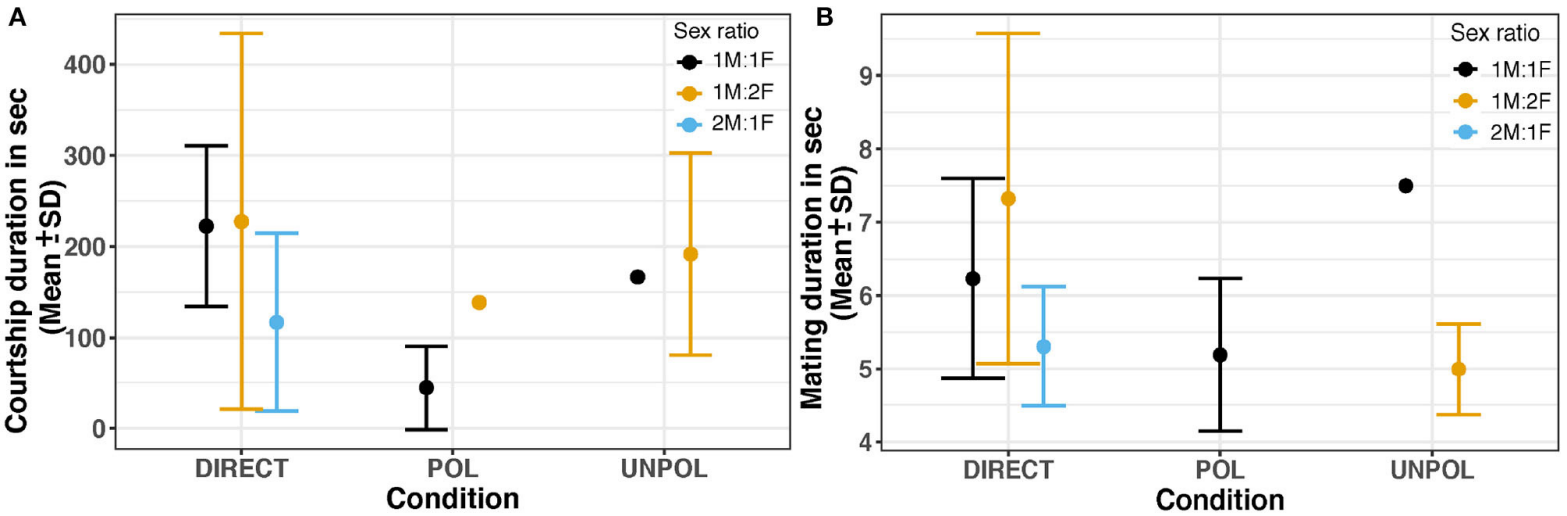
**(A)** Courtship duration in second. **(B)** Mating duration (s). Large dots and bars represent the mean and standard deviation, respectively. DIRECT, Control (no barrier); POL, Polarized Barrier; UNPOL, Unpolarized barrier. Sex ratio = 1M:1F in black, 1M:2F in yellow, 2M:1F in blue.

Courtship duration was highly variable as males exhibited courtship displays between one to eight times (Figure 4A). The shortest courtship was 11.75 sec, and the longest was 1867.35 sec, with a mean of 161.22 ± 106.82 sec. We analyzed courtship duration with Factorial ANOVAs using transformed data (as the relative percentage from total duration (%), and also z-scores); however, the sex ratio and types of barriers did not have a significant effect on courtship duration (*p* > 0.05) (Figure 5A).

We described the DPS as a new pattern (see Supplementary Video 1), which was observed in three 1M:1F - control tests, one 1M:2F - control experiment, and one 2M:1F - unpolarized test. The duration of DPS was 191.101 ± 353.20 sec. Males displaying DPS pattern had a high success rate of mating, as four males mated using this particular display (80.00%).

The agonistic signal with intense zebra coloration had a duration of 930.14 ± 1144.17 sec. This pattern was present in 19 control experiments (57.58%), one POL (3.03%), and three UNPOL observations (9.09% from all the trials). Cuttlefish exhibited shovel displays only in six control trials and had duration 190.38 ± 290.26 sec. Only two cuttlefish showed the lateral display pattern, with a duration of 36.255 ± 49.15 sec in two observations (2M:1F - control). Four cuttlefish showed mate-guarding behavior with duration 289.07 ± 288.04 sec in 2M:1F control experiments.

The cumulative mating duration was between 4.35 and 24.826 sec, with a mean of 10.31 ± 0.66 sec. Statistical analysis did not reveal any significant differences in mating duration (*p* > 0.05) among the sex ratios and polarized conditions tested in our study (Figure 5B).

### 3.4. Differences in Type and Sequence of Body Patterns Between Successful and Non-successful Courtships

**Figure 6:**
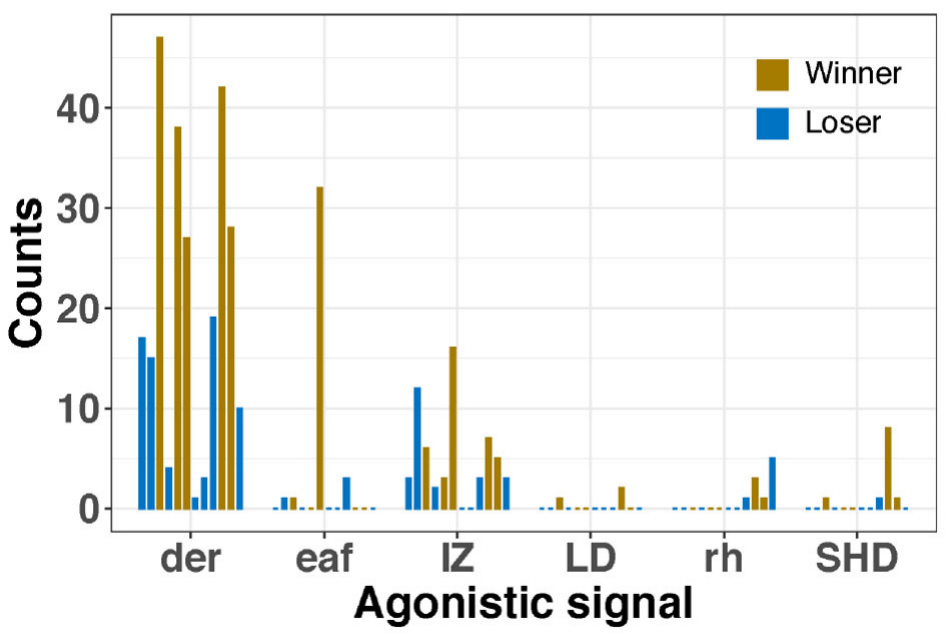
Frequency of each agonistic signal by every male during male competitions. Dark eye ring (der), extended arm IV (eaf), Intense Zebra pattern (IZ), lateral display (LD), raised head (rh), and shovel display (SHD). Each bar represents a male and the number of times each signal was observed. Winners in yellow and losers in blue.

**Figure 7:**
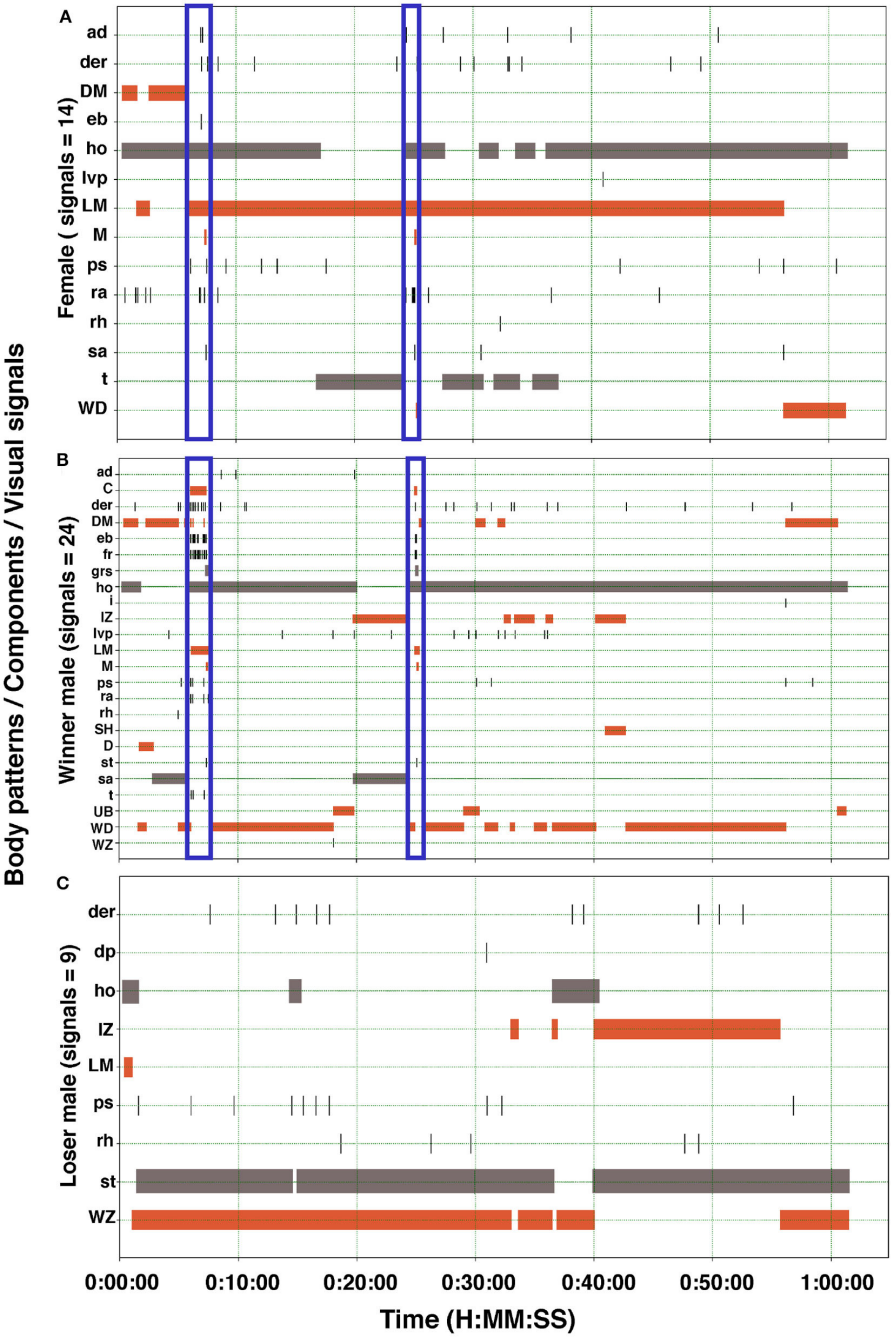
Ethogram of the body patterns and behaviors of two males and a female *S. plangon* showed for courtship and mating. The orange horizontal bars represent duration of the chronic patterns. Gray bars denote state events. Black vertical lines correspond to point events and acute patterns. Blue vertical rectangles encompass signals during courtship and mating. **(A)** In this observation, the female displayed 14 visual signals (Body patterns and components). **(B)** Winner male displayed up to 24 signals, whereas the loser male **(C)** showed only nine. See Table 1 for the codes' abbreviations.

**Figure 8:**
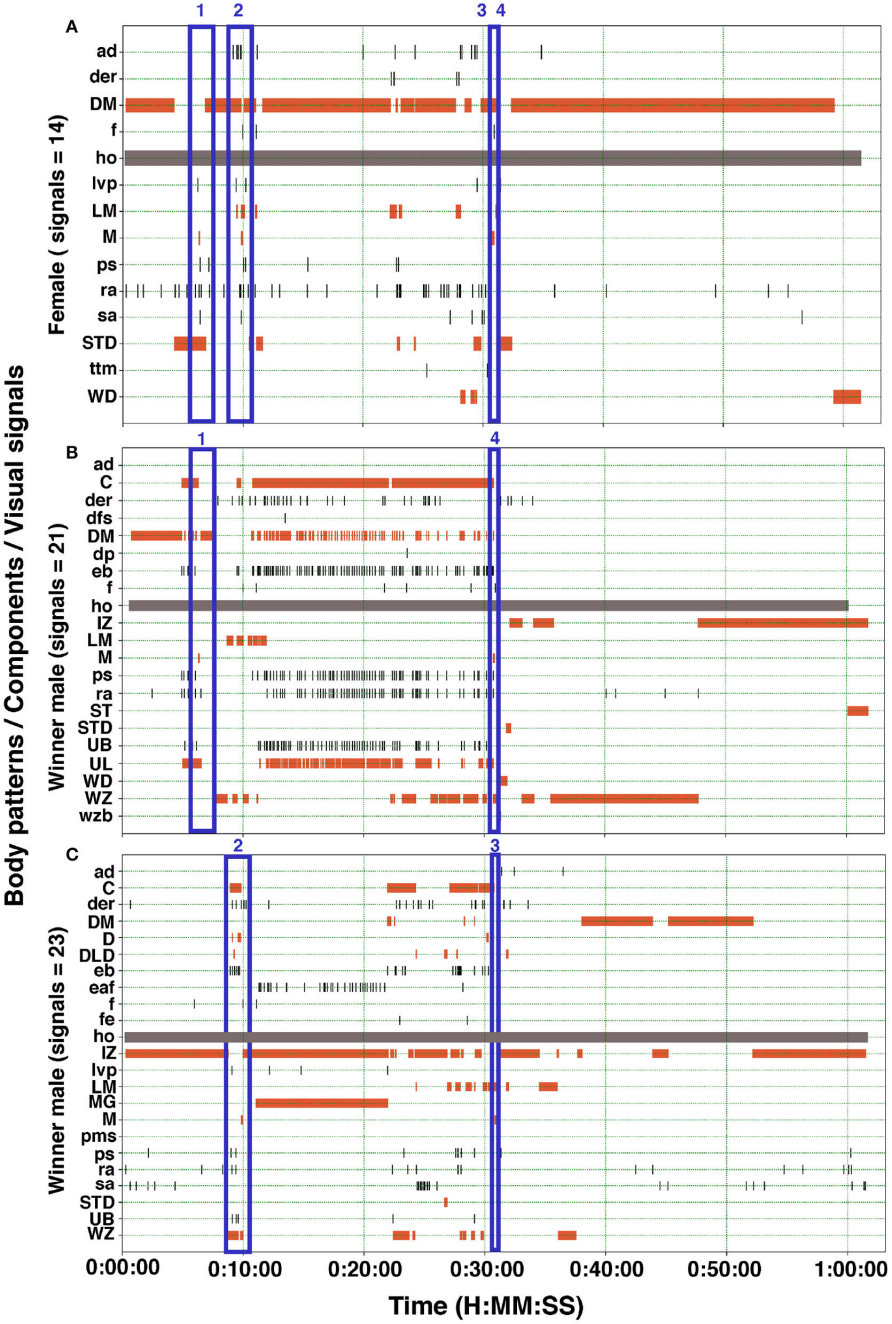
Ethogram of the body patterns and reproductive behaviors of two males and a female *S. plangon*. In this experiment, the female **(A)** mated twice with each male **(B,C)**. The orange horizontal bars represent duration of the chronic patterns. Gray bars denote state events. Black vertical lines correspond to point events and acute patterns. Blue rectangles encompass signals during courtship and mating. The numbers above the blue rectangles represent the mating events. Mating 3 and 4 occurred very near to each other. See Table 1 for the codes' abbreviations.

**Figure 9:**
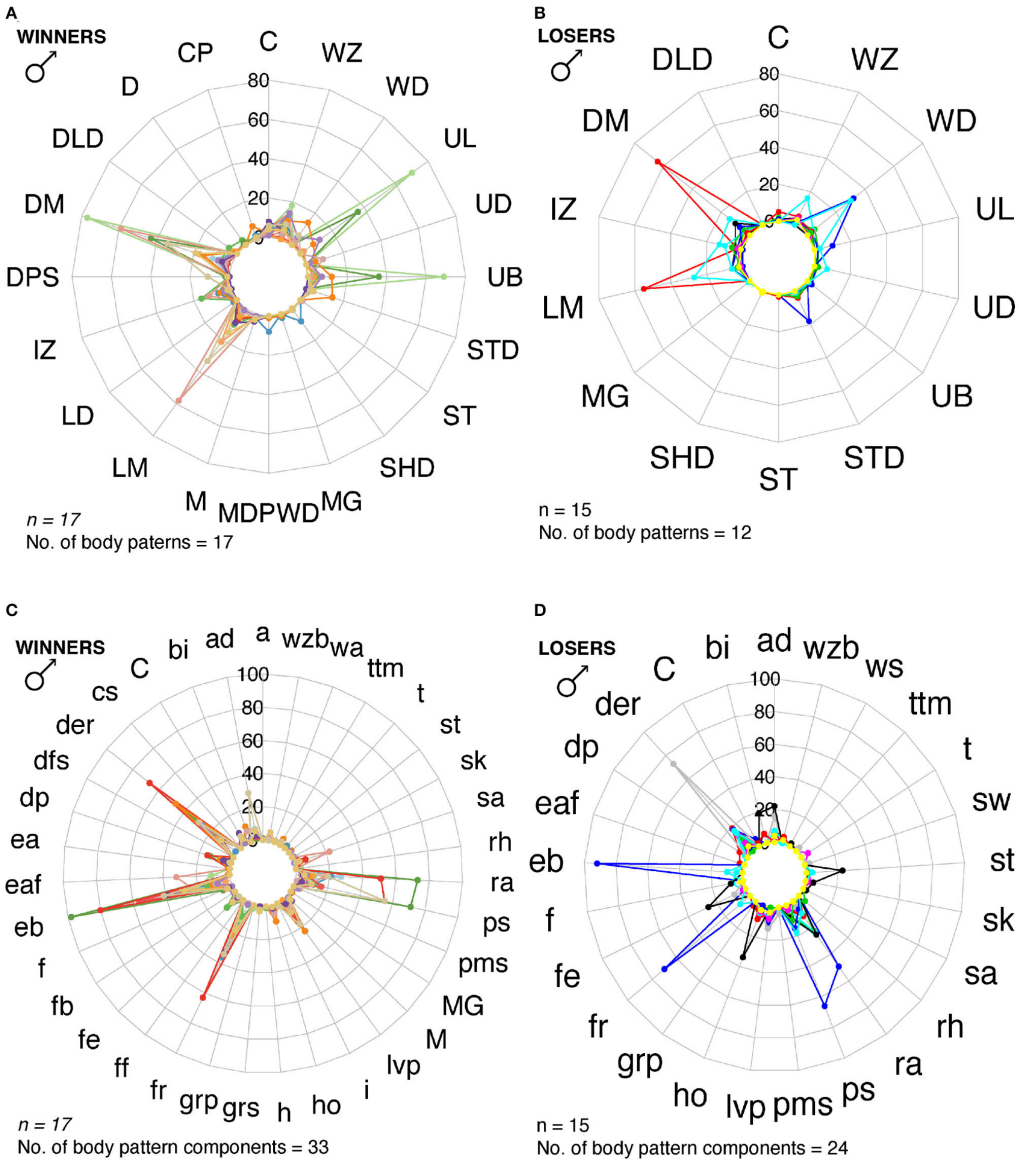
Radar charts representing the number of body patterns and components that winner and loser males used for reproductive behavior. Each colored line corresponds to a different individual. **(A)** Winner males (*n* = 17) showed a max of 17 body patterns. **(B)** Winner males showed 33 body pattern components. **(C)** Loser males (*n* = 15) displayed a max of 12 body patterns, and 24 body pattern components **(D)**. M, Mating; MG, Mate Guarding; C, Courtship. See Table 1 for the codes' abbreviations.

Cuttlefish displayed up to 17 body patterns during courtship, agonistic fights, mate guarding, and copulation. Males showed a specific sequence of body patterns for courtship display (Figure 3), including light mottle, intense zebra, weak zebra, dark mottle, uniform blanching, uniform darkening, and dark mottle. Rapid changes between smooth and papillate skin, elongated and flattened body, extended arms, forward rush, dark eye rings, and turning toward the female were also part of courtship displays. Males that mated at least once displayed up to 17 body patterns and 33 body pattern components (Figures 6, 7B, 8B,C, 9A,C); whereas loser males exhibited a maximum of 12 body patterns, and 24 components (Figures 7C, 9B–D). The most frequent signals amongst winner males were dark eye ring, elongated body, forward rush, raised arms, and papillate skin, dark mottle, light mottle, uniform blanching, and uniform light (Figures 9A,C).

**Figure 10:**
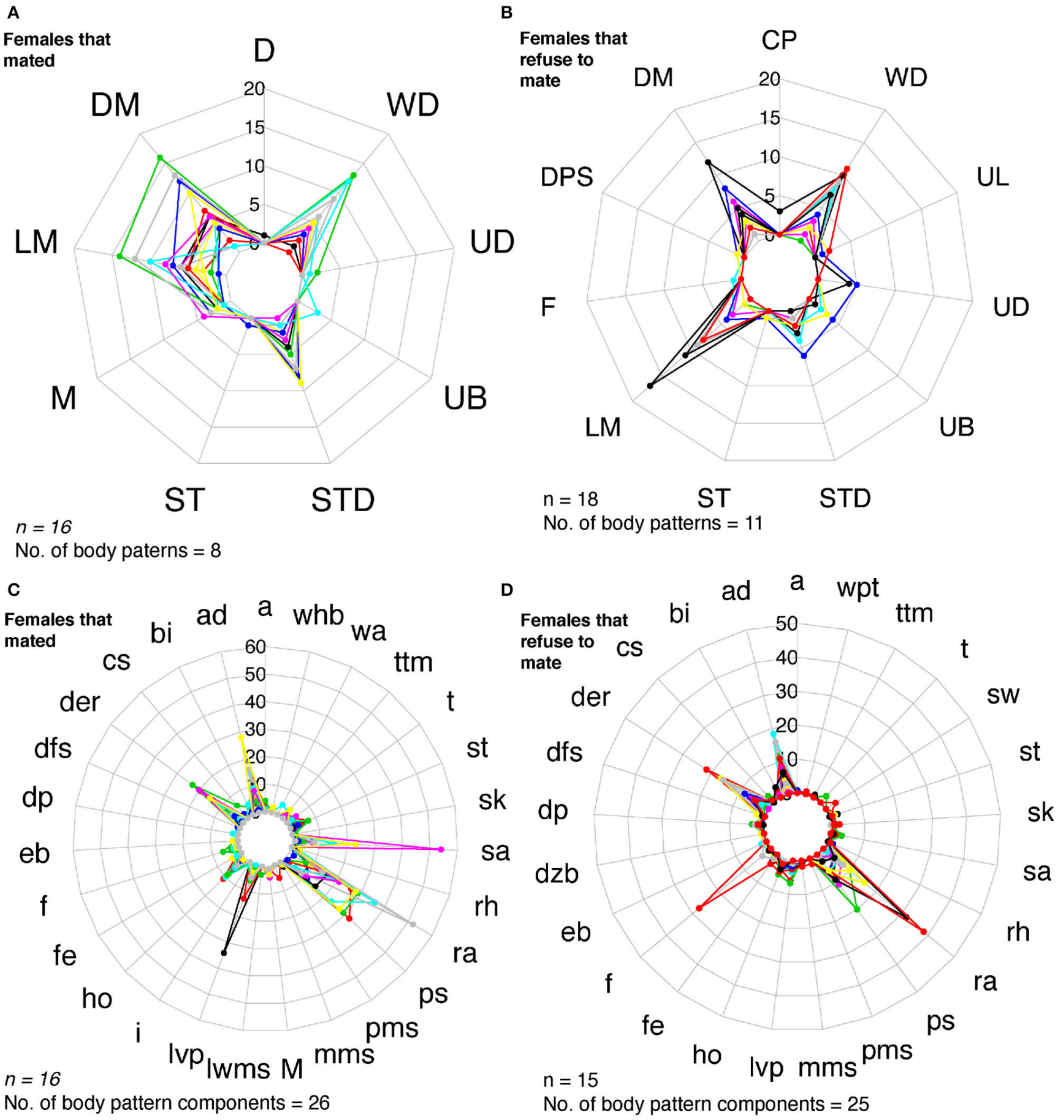
Radar charts representing the number of body patterns and components of females that chose to mate and females that did not. Each colored line corresponds to a different individual. **(A)** Females that opted to mate (*n* = 16) showed up to eight body patterns and **(B)** 26 body pattern components. **(C)** Females that refused to mate (*n* = 18) exhibited 11 body patterns, and 25 body patterns components **(D)**. M, Mating. See Table 1 for the codes' abbreviations.

**Figure 11:**
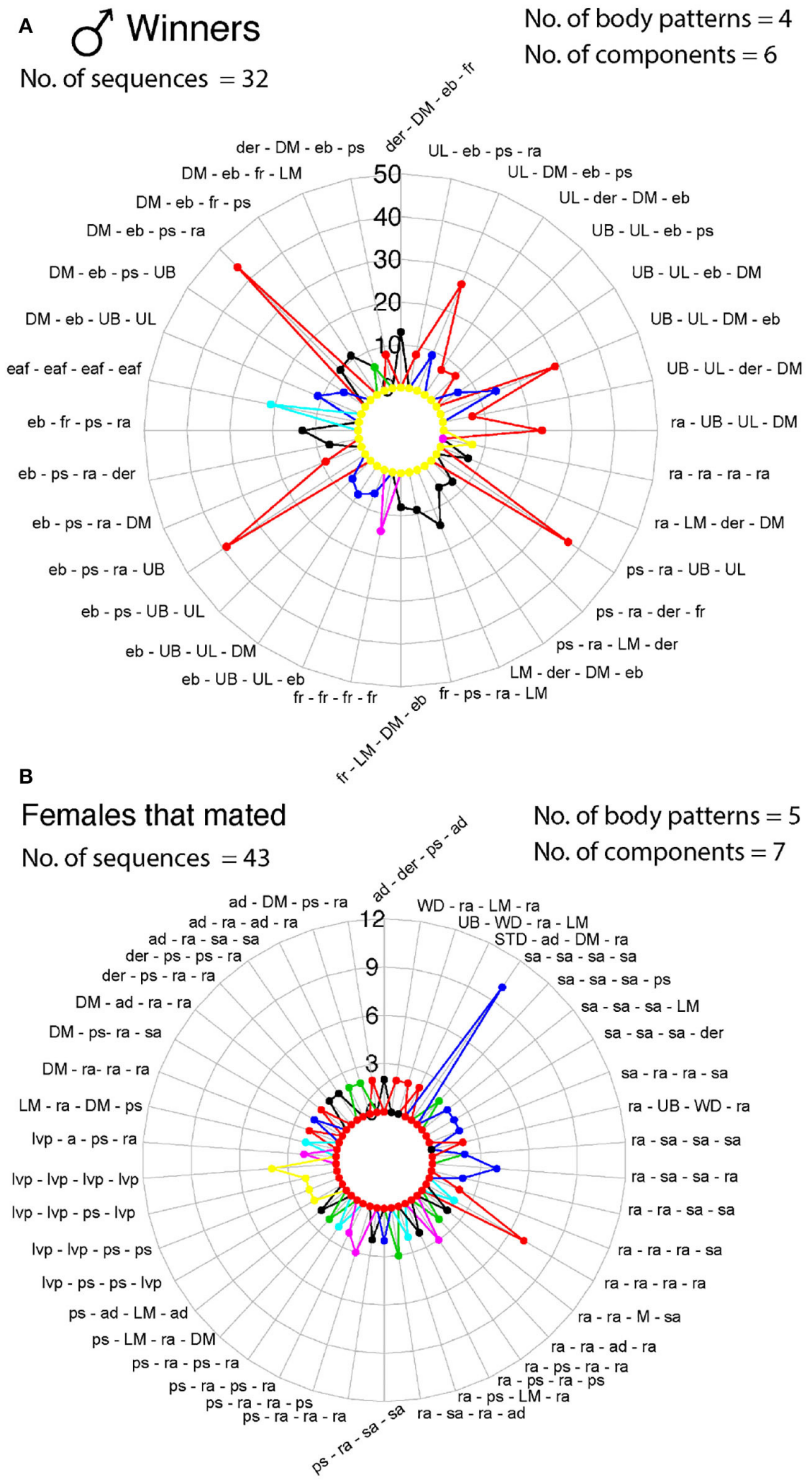
Radar charts representing the sequence of signals (body patterns and components) that *S. plangon* used for mating. Each colored lined represents a cuttlefish. Each sequence was observed more than twice in all cuttlefish. **(A)** Winner males. A total of 32 sequences were observed in males that successfully mated. Combinations of four body patterns (DM, LM, UB, and UL) and six components (der, eb, ps, fr, eaf, and ra) were observed in all sequences. **(B)** Females that were inclined to mate exhibited up to 43 sequences; however, the frequency of these signals was lower than those in males. Five body patterns (DM, LM, UB, WD, and STD), and seven components (a, ad, der, ps, ra, sa, lvp) were showed by the females for these sequences. See Table 1 for the codes' abbreviations.

We identified 32 sequences of components and body patterns that were relevant for successful mating. Males showed these sequences for at least five times throughout courtship displays. These sequences were made of combinations between one textural component (ps), a locomotor component (fr), two postures (eaf, ra), one chromatic component (der), and four patterns (DM, UB, UL, and LM) in an orderly fashion (**Figure 11A**). Losers showed a similar sequence of body patterns and components; however, the frequency of these signals was much lower than those displayed by winner males (Figure 6). On the other hand, females showed 43 visual signal sequences before mating. One textural component (ps), one locomotor (a), three postural (ad, ra, sa), two chromatic (der, lvp), and five patterns (DM, LM, UB, WD, STD) encompassed the sequences in females for mating (Figures 10A,C, 11B). On the other hand, females that refused to mate kept the arms dropped, dark eye rings, fled and showed papillate skin as response to the courtship displays of the males (Figures 10B,D).

**Figure 12:**
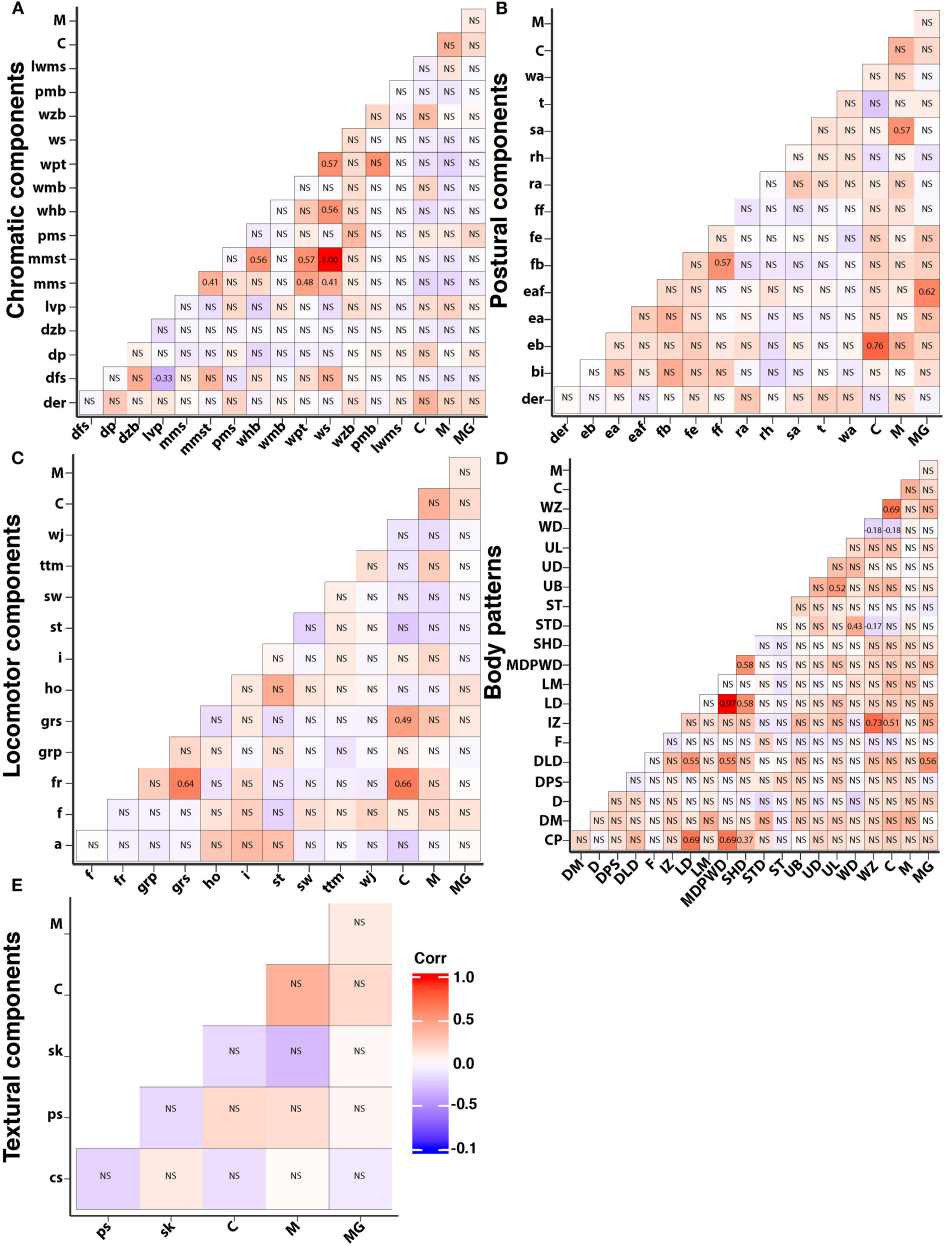
Heatmap with pairwise correlations between all the body patterns, components, and courtship (C), mating (M), mate guarding (MG) of *S. plangon*. Red squares are positive correlations and blue are negative. Non significant *p* values are marked with NS. **(A)** Chromatic components. **(B)** Postural components. In this case, the only component correlated to courtship was elongated body. Similarly, splayed arms was significantly correlated to mating, and extended IV arm to mate guarding. **(C)** Locomotor components. Courtship was significantly correlated to forward rush and grasping. **(D)** Intense zebra, weak disruptive, and weak zebra were significantly correlated to courtship. **(E)** All Skin texture components were non-significant. See Table 1 for all the codes' abbreviations. Significant *p*-values were marked with their corresponding coefficients of determination (*r*^2^) in black numbers.

We integrated the data from all subjects into a matrix with the frequency of each behavior, body pattern, and component to analyze the correlation between them (Figure 12). We found that courtship was significantly correlated to forward rush (*r*^2^=0.66, *p* < 0.01), grasping (*r*^2^ = 0.49, *p* < 0.01), elongated body (*r*^2^=0.66, *p* < 0.01), Intense Zebra pattern (*r*^2^=0.51, *p*=0.01), Weak Disruptive (*r*^2^=−0.183, *p*=0.042), and Weak Zebra pattern (*r*^2^=0.69, *p* < 0.01). Mating was significantly correlated to splayed arms only (*r*^2^=0.57, *p* < 0.01); while extended IV arms (*r*^2^=0.62, *p* < 0.01) and Dual-Lateral Display (*r*^2^=0.56, *p* < 0.01) were significantly correlated to mate guarding.

### 3.5. 3D Printed Cuttlefish

We analyzed the behavioral responses of the cuttlefish in the presence of one or two dummies (number of experiments, *n* = 8). However, cuttlefish did not attempt to mate with the dummies. *Sepia plangon* displayed defense signals, such as deimatic, dark mottle, strong disruptive, weak zebra, flee, hiding, inking, dilated pupils, and papillate skin toward the dummies, or remained away from them. No agonistic behavior was observed in these experiments.

## 4. Discussion

This study is the first to describe in detail the mating behavior of *S. plangon* under different light conditions and sex ratios in captivity. The intricate mating system of *S. plangon* comprises male agonistic behavior and signaling, alternative reproductive tactics, female mate choice, and multiple matings. Furthermore, the temporal order of component and body pattern expression is critical to winning mating competitions, similar to the communication system in the reef squid (Lin et al., 2017).

In the spawning season, *S. plangon* showed strong sexual dimorphism as mature females were larger than males. Similar results were previously reported by Beasley et al. (2017). Larger females are relatively common in other cephalopod species, for example, in the squid *Dosidicus gigas* (Nigmatullin et al., 2001), the octopus *Eledone cirrhosa* (Regueira et al., 2013), and *Haliphron atlanticus* (Lu and Chung, 2017). On the other hand, *S. plangon* male size might be a critical factor that lead to the development of male alternative mating strategies similar to those in squids (Wada et al., 2005; Lin and Chiao, 2018), and octopus (Huffard et al., 2008). For instance, smaller males can use different mating behavior (e.g., male-upturned and sneaking) to avoid male competition and mate. In this study, we observed small *S. plangon* males using the dual-lateral display to avoid male competitions and mate with large females. Similar results were previously reported by Brown et al. (2012).

In our study, we observed that males *S. plangon* were smaller than females; therefore, it is likely that the body size does not determine male dominance in this species while in others such as *S.apama* it does (Hall and Hanlon, 2002). The number and variety of displays potentially act as signals to communicate male fitness, which could influence *S. plangon* female choice. Other investigations have shown that larger females generally have higher fecundity and produce larger offspring in mammals (Kilanowski and Koprowski, 2016), insects, and arthropods (Honěk, 1993; Fox and Czesak, 2000; Stillwell et al., 2010). Additionally, intersexual selection may drive the evolution of small male size in *S. plangon*, for example, small body size could be beneficial to males that show dynamic or acrobatic courtship (Székely et al., 2004).

### 4.1. Courtship Behavior, Agonistic Signals, and Mating

Sexually selected signals fall into two categories, signals used in inter-sexual displays (e.g., courtship), or signals used in intra-sexual displays (agonistic signals) as proposed by Andersson (1994). Courtship includes one or more sensory modalities (visual, olfactory, auditory, tactile, and some others), and often leads to the evolution of traits through sexual selection (Owren et al., 2010). Precopulatory processes occur in both females and males, such as male-male competitions, female and male mate choice (Kuester and Paul, 1996; Johannesson et al., 2008; Edward and Chapman, 2011; Hamel et al., 2015; Gwynne, 2016; Roberts and Mendelson, 2017). Postcopulatory mechanisms are sperm competition in males (Simmons, 2014), cryptic female choice (CFC), and cryptic male choice (CMC). CFC occurs when females use specific traits or mechanisms to influence the probability that males fertilize their eggs, whereas CMC is any male behavior that allows males to bias their investment in matings toward certain females (Eberhard, 1996; Reinhold et al., 2002; Arnqvist, 2014).

Previous literature has not found clear evidence of courtship displays in some cephalopods species, such as *S. apama* (Hall and Hanlon, 2002), *S. officinalis* (Boal, 1997; Adamo et al., 2000), *Idiosepius paradoxus* (Kasugai, 2000; Sato et al., 2010), and *Euprymna scolopes* (Hanlon et al., 1997). Nonetheless, intricate courtship displays have been described in *S. latimanus* (Corner and Moore, 1980; Hanlon and Messenger, 2018), and *S. sepioidea* (Moynihan and Rodaniche, 1982), and *Loligo pealei* (Hanlon, 1996). These patterns are characterized by quick changes in patterns and bright colorations (Hanlon and Messenger, 2018). It is notable in *S. plangon* that the chromatic changes described are in fact largely a-chromatic, that is black and white and most likely signaling in contrast, not color.

Males *S. plangon* potentially established dominance through the use of visual signals (Figure 6), such as intense zebra, extended IV arms, uniform blanching, dark eye rings, lateral display, uniform darkening, shovel display, and elongated body. Escalation to physical fights was only observed in one control experiment (2M:1F) and had a duration shorter than 6 s. Similar results were observed in the giant cuttlefish *S. apama* (Hall and Hanlon, 2002; Schnell et al., 2015b) and the squid *Loligo plei* (DiMarco and Hanlon, 1997), as males contest duration and frequency decreased by the presence of a female and whether temporary pairing had occurred. By contrast, smaller *S. plangon* males (ML < 80mm) showed the dual-lateral display (DLD) to sneak in and mate with females without fighting with a dominant male. Our results suggest that DLD is an efficient tactic that small cephalopod *S. plangon* use to avert a fight with larger rivals and obtain opportunities to mate. Similar cases were reported in *S. plangon* by Brown et al. (2012), the squid *Sepioteuthis sepioidea* (Hanlon and Messenger, 2018), and *S. apama* (Hall and Hanlon, 2002; Naud et al., 2004; Hanlon et al., 2005). Males mimicking females are also commonly observed in birds, lizards, and crustaceans. For instance, male pied flycatchers use female mimicry as an advantage to choose when to initiate an attack, thus increasing the chances of winning male contests (Saetre and Slagsvold, 1996). Males Augrabies flat lizards often mimic female coloration to avoid the injuries and energetic cost associated with fighting other males; however, they still use male pheromones as an honest signal of their gender for mating (Whiting et al., 2009). Spider crabs (Laufer and Ahl, 1995), and isopods (Shuster, 1989; Shuster and Wade, 1991) also use female mimicry as an alternative mating tactic to access females and avoid male competitions.

In our study, we observed four male cuttlefish mate-guarding the females only in 2M:1F control experiments. The guarded females were large, fully mature, carrying eggs, and had ML between 73.00 and 90.00 mm. We did not determinate the number of eggs carried by the guarded females; however, this strategy could represent a cryptic male choice, as males could bias their mate-guarding efforts toward particular females (Aumon and Shuker, 2018). The bobtail squid *Sepiadarium austrinum* exhibited strategic male choice as their mating efforts were more substantial toward egg-carrying females (Wegener et al., 2013; Hooper et al., 2016). Similarly, large male *Abdopus aculeatus* copulate frequently in mate-guarding situations with large females Huffard et al. (2008). Several studies have reported temporary mate guarding in several species of cephalopods, such as *S. apama* (Hall and Hanlon, 2002; Naud et al., 2004), *S. officinalis* (Adamo and Hanlon, 1996; Hanlon and Messenger, 2018), and *Loligo pealeii* (Shashar and Hanlon, 2013). Precopulatory mate guarding might allow the male to monopolize the female until she is receptive, and postcopulatory mate guarding could prevent females from prematurely removing the sperm and ensure insemination.

We observed only one female mating with both males; however, fifteen females had multiple matings (Figures 7A, 8A), and nineteen females did not mate once. Possibly, potential pre and postcopulatory CFC also occur in *S. plangon*, as some females rejected mating attempts, but others had multiple matings with several males. Potentially, females *S. plangon* could choose the sperm that fertilizes their eggs. Previous investigations have analyzed CFC in squids (Sato et al., 2013, 2014, 2016; Shashar and Hanlon, 2013; Mather, 2016; Lin and Chiao, 2018; Iwata et al., 2019), and octopus (Huffard et al., 2008; Morse et al., 2015; Morse and Huffard, 2019); however, CFC studies in cuttlefish are limited (Boal, 1997; Hall and Hanlon, 2002; Naud et al., 2005). Boal (1997) found that females *S. officinalis* prefer to mate with males that had copulated recently. According to Hall and Hanlon (2002), *S. apama* might possess a mechanism for postcopulatory CFC. Two sources of sperm were available to the female to fertilize the eggs: (1) spermatangia from the most recent matings around the buccal region, and (2) sperm stored internally in receptacles located around the beak. Similar results were reported by Naud et al. (2005) in *S. apama* using genetic analysis. They found evidence supporting the biased use of sperm from those sources mentioned above, which suggests a potential postcopulatory CFC in this species. We collected animals from the wild and did not control whether females had already mated, which could reduce mating likelihood in our experiments. Future studies should focus on both female and male cryptic choice, comparing the probability of mating with virgin cuttlefish, and analyze whether females choose the sperm to fertilize the eggs from one male or another (Iwata et al., 2019).

The sex ratio was the only factor affecting courtship frequency in *S. plangon* of the two factors tested in our experiments. On the other hand, the intensity and frequency of male competitions in *S. plangon* were not affected by sex ratio. Similar results were reported in flies (Leftwich et al., 2012), and fish (de Jong et al., 2009; Clark and Grant, 2010), as the sex ratio had a significant effect on the courtship behavior and duration. Lobsters (Debuse et al., 1999), fish (Mills and Reynolds, 2003), and arthropods (Enders, 1993; Waiho et al., 2015) change the reproductive behavior depending on the sex ratio. For instance, at high male density (more than three males) large European bitterling males ceased to be territorial and instead competed with groups of smaller males (Mills and Reynolds, 2003). It is likely that our test did not trigger frequent aggressive male fights because we only placed two males with one female as the highest sex ratio for males.

POL and UNPOL barriers did not have a significant effect on the frequency and duration of courtship, agonistic encounters, and mating. However, the POL barrier caused a large variation in courtship and mating frequency (Figure 4). Likely, GLM did not find any statistical significance because we have more observations in control experiments than POL-UNPOL tests; thus, statistical power could be a limitation in our study. Although POL and UNPOL barriers limited the physical contact between cuttlefish and modified the light conditions, males started their repetitive courtship display. Several studies have suggested that polarized light is used in cephalopods for communication and navigation (Shashar and Cronin, 1996; Shashar et al., 2000; Boal et al., 2004; Saidel et al., 2005; Chiou et al., 2007; Talbot and Marshall, 2010b; Cartron et al., 2013a; Marshall et al., 2019); however, to date, there is no conclusive evidence that shows the function of polarization signals in the reproductive context. This study showed that changes in polarized light did not affect mating behavior in *S. plangon*, and that the presentation and sequence of body patterns were decisive for mate choice.

### 4.2. Visual Signaling and Communication

The body patterns of *S. plangon* are similar to those of *Sepia officinalis* (Hanlon and Messenger, 1988), *Sepia pharaonis* (Nakajima and Ikeda, 2017), and *M. pfefferi* (Roper and Hochberg, 1988; Thomas and MacDonald, 2016). We identified 18 body patterns in mature male and female *S. plangon*, whereas *S. officinalis* and *S. pharaonis* have only 13 (Hanlon and Messenger, 1988; Nakajima and Ikeda, 2017). Our study used mature individuals, whereas the descriptions of *S. officinalis, S. pharaonis*, and *M. pfefferi* were based on juveniles. Additionally, our study revealed signals and body patterns used for reproductive behavior, such as multidirectional passing wave display, shovel display, lateral display, and dynamic polarization signals which were not reported in the studies of Hanlon and Messenger (1988), Thomas and MacDonald (2016), and Nakajima and Ikeda (2017).

Two patterns in *S. plangon* are rare in other cephalopod species: the dual lateral display (DLD), and the dynamic polarization signals (DPL). DLD was previously described in males *S. plangon* by Brown et al. (2012), and we also identified this pattern in five small males (ML < 80.00 mm) that were between a larger rival and a female. DLD is a deceptive signal, and similar to *S. sepioidea* (Hanlon and Messenger, 2018), and *S. latimanus* (Corner and Moore, 1980), is often used as an alternative method to avoid competitions and find females to mate. This behavior is not particular to cephalopods; male cricket frogs change their dominant calls in the presence of an opponent to mimic the female calls (Wagner, 1992). Likewise, females dance fly can also use deceptive signals to prey on males seeking for mates (Funk and Tallamy, 2000). The evolutionary consequences of deceptive displays are hard to interpret as they depend on the costs and benefits of deception to both senders and receivers (Stuart-Fox, 2005). We reported 80.00% of mating success in males *S. plangon* that used DLD to avoid male competitions; possibly, DLD is a common and successful strategy in the mating system of *S. plangon* (Brown et al., 2012).

We reported DPL as a new pattern in our study. This display was exclusive to males and used during courtship, and involved running bands across areas where cuttlefish reflected strong PL signals; four of five males successfully mated after showing this pattern. This pattern is similar to the Passing cloud display of *S. officinalis* (Hanlon and Messenger, 1988). However, the passing cloud was only reported in juveniles, and it is a defense mechanism involving bands running across the entire body. In contrast, DPS has dark bands running horizontally across the arms stripes and around the eyes. It is possible that *S. plangon* might control the intensity of PL signals for courtship by controlling the expansion and retraction of chromatophores as dark bands over the arms stripes. Although the evidence supporting the direct control of PL signals for communication in cuttlefish is not definitive yet (Shashar et al., 1996, 2002; Mäthger et al., 2009; Marshall et al., 2019), Gonzalez-Bellido et al. (2014) reported that the expression of iridescence in squids was controlled by the brain but also changed in response to environmental luminance. Thus, the iridophores in cuttlefish reflect strong PL signals potentially controlled by the brain, and these signals could be used for communication with conspecifics (Shashar et al., 1996). In this study we did not find any effect by POL and UNPOL barriers; however, the perspective of the animal under natural conditions should be considered in the future to investigate polarized vision and communication (Marshall et al., 2019).

*S. plangon* body patterns differed between the winner and loser males. Winners showed up to 17 body patterns and 33 components, whereas losers only showed 12 patterns and 24 components. Conversely, females that did not mate showed more body patterns (18) than mating females (16) (Figure 10). The dynamic and repetitive nature of the courtship displays was similar between winners and losers; however, the number of times that winner showed each pattern and component of the courtship was higher than those in loser (Figure 4). Highly repetitive signals may have provided more information about mate quality by transmitting the same message (courtship display) multiple times. Therefore, females could assess more accurately one or more stimuli that are displayed repeatedly before choosing a mate (Mowles and Ord, 2012). We identified 32 sequences of visual signals displayed by males *S. plangon* that were crucial for successful mating. These sequences were composed of combinations of four body patterns (DM, LM, UB, and UL) and six components (der, eb, ps, fr, eaf, and ra) in a specific order (Figure 11). Similarly, females showed up to 43 sequences composed of five body patterns (DM, LM, STD, UB, and WD) and seven components (a, ad, der, lvp, ps, ra and sa). Therefore, it was not just the presentation of these body patterns that led to mating, but the sequence of specific body patterns. A similar study done by Lin et al. (2017) analyzed the visual signals and body patterns of the squid *S. lessoniana* for reproductive behavior. They reported that each behavior was composed of multiple chromatic components, and each component is often involved in multiple behaviors. Thus, the dynamic body patterning and expression of unique sets of components represents an efficient communication system in squids. Our results suggest that *S. plangon* also use specific set of signals (body pattern, chromatic, postural, locomotor, and textural components) to communicate efficiently for successful mating. In females, the most frequent postural components for mating involved the arms (e.g., arms dropped, raised arms, and splayed arms), which could be associated with the fact that male cuttlefish deposit the sperm in the female's buccal area (Hall and Hanlon, 2002; Naud et al., 2004; Hanlon and Messenger, 2018). Therefore, females exposing the buccal area to the male could be interpreted as a positive cue for mating.

The dummies used in this study did not trigger behavioral interactions with the cuttlefish, suggesting that a static body pattern component is not a strong stimulus to start courtship behavior. One suggestion for future studies would be to present videos of real animals to the cuttlefish and see whether the video of a real mate triggers courtship behavior. In fact, Pignatelli et al. (2011) and Temple et al. (2012) have previously shown that squids and cuttlefish react to computer-generated polarized looming stimuli. However, these investigations did not test the reaction to a PL video of a real cuttlefish displaying courtship patterns.

This study was the first to report in detail the reproductive behavior of *S. plangon* under different sex ratios and light conditions. Sex ratio was the only factor that had a significant effect on courtship frequency. Furthermore, we showed evidence that the size or presentation of a specific body pattern and posture is not sufficient to initiate courtship behavior in *S. plangon*, as 3D models did not trigger any mating. We found that the number and specific order of sequences of body patterns and components are determinant for successful matings, presented as dynamic courtship signals. We introduced *S. plangon* as an attractive animal model and very convenient for laboratory behavioral studies due to its small size.

## Data Availability Statement

The original contributions presented in the study are included in the article/supplementary files, further inquiries can be directed to the corresponding authors.

## Ethics Statement

The animal study was reviewed and approved by the Animal Ethics Unit, Office of Research Ethics of the University of Queensland (Permit number QBI/304/16).

## Author Contributions

AL performed the experiments, analyzed the data, and drafted the manuscript. All authors contributed to the design of experiments, manuscript revision, and approved the final version.

## Conflict of Interest

The authors declare that the research was conducted in the absence of any commercial or financial relationships that could be construed as a potential conflict of interest.

## Supplementary Material

Supplementary Video 1 The flickering effect in the video represent the areas where *Sepia plangon* reflect polarized signals. The video was recorded with a modified Sony©HVR-Z1P video-camera. The camera had a nematic switch-plate polarizer attached. Alternate horizontal (H) and vertical (V) polarizing were fitted every other frame.

Supplementary Video 2 Video from a polarization camera. Left: intensity (black and white image). Center: degree (%) polarization, scale 0–100%, blue to white with deep-red at approximately 45%. Right: angle or e-vector direction, the circular key shows orange/red as horizontal and cyan as vertical. Video from a mature female *Sepia plangon*.

Supplementary Video 3 Two cuttlefish *Sepia plangon* during courtship behavior. Male (top) showing dynamic polarization signals (DPS) to the female (bottom). This pattern appeared as moving bands and flashes over the arms stripes and eye sclera, which are areas with strong polarized reflection (see Supplementary Video 2).

Supplementary Figure 1 Patterns and behaviors observed during courtship, agonistic, and mating in *S. plangon*. **(A)** Two males and a female. To the right, male showing shovel display (SHD) as agonistic signal to the other male. **(B)** Two males fighting for the female. Males showed Intense Zebra, or Dark Mottle coloration, dark eye rings, and extended Arms to push the competitor. Meanwhile, the female hold a dark mottle coloration. **(C)** A large male with intense zebra pattern pushing a small male away from the female. The small male showed light mottle pattern. **(D)** Close view of a male showing DLD, two patterns simultaneously (intense zebra and dark mottle). **(E)** “Sneaker” male showing dark mottle pattern to the other male, and intense zebra to the female. **(F)** A male and a female adopting the mating position in a experiment with a polarized barrier between cuttlefish. The barrier was attached to PVC pipes.

Supplementary Figure 2 **(A)** Mantle length (ML) and **(B)** Total length (TL) of mature females (gray boxplot, *n* = 34) and males (yellow boxplot, *n* = 32) *S. plangon*.

## Appendix

### Material Methods

#### Sample Size

**Table A1:** Sample size (*n* = number of experiments) for each condition tested in this study.

	**1M:1F- DIRECT**	**1M:1F- POL**	**1M:1F- UNPOL**	**1M:2F- DIRECT**	**1M:2F- POL**	**1M:2F- UNPOL**	**2M:1F- DIRECT**	**2M:1F- UNPOL**
n	6	2	1	5	2	2	6	1

### 3D Printed Cuttlefish Models

- CGTrader model 1: https://www.cgtrader.com/3d-models/animals/fish/cuttlefish-e0177629-4eba-4166-b54c-a2b06c9e011c
- CGTrader model 2: https://www.cgtrader.com/3d-models/animals/fish/low-poly-cuttle-fish-animated-game-ready
- CGTrader model 3: https://www.cgtrader.com/3d-models/animals/fish/cuttlefish-19d89111-3607-4770-8303-59f5a98c2dde.
