# Supplementary figures and images for: Dynamic Courtship Signals and Mate Preferences in Sepia plangon
Source: Front Physiol. 2020 Aug 7;11:845. doi: 10.3389/fphys.2020.00845 (PMC7438932; doi:10.3389/fphys.2020.00845)

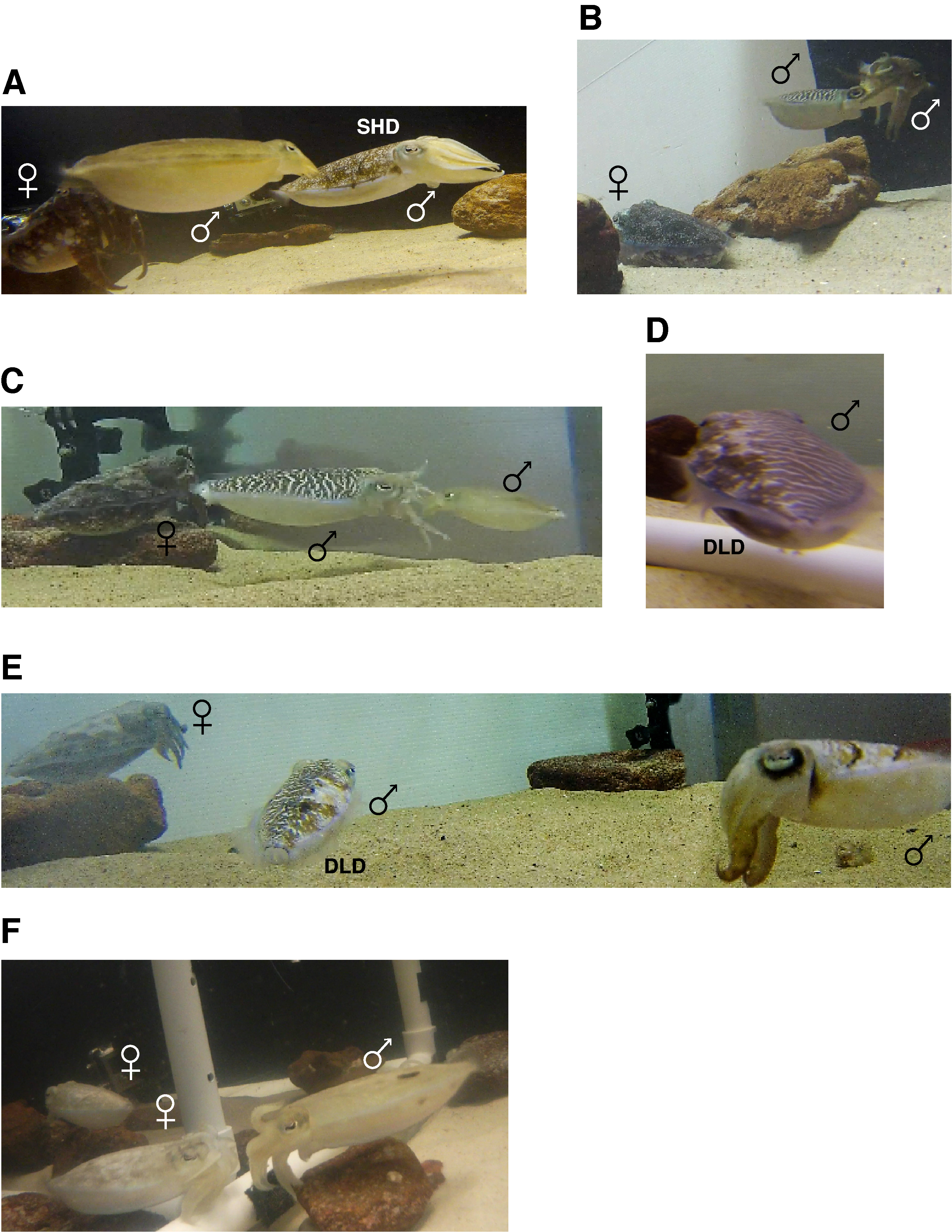

Supplement: Supplementary Figure 1 — Patterns and behaviors observed during courtship, agonistic, and mating in S. plangon. (A) Two males and a female. To the right, male showing shovel display (SHD) as agonistic signal to the other male. (B) Two males fighting for the female. Males showed Intense Zebra, or Dark Mottle coloration, dark eye rings, and extended Arms to push the competitor. Meanwhile, the female hold a dark mottle coloration. (C) A large male with intense zebra pattern pushing a small male away from the female. The small male showed light mottle pattern. (D) Close view of a male showing DLD, two patterns simultaneously (intense zebra and dark mottle). (E) “Sneaker” male showing dark mottle pattern to the other male, and intense zebra to the female. (F) A male and a female adopting the mating position in a experiment with a polarized barrier between cuttlefish. The barrier was attached to PVC pipes. [file Image_1.JPEG]

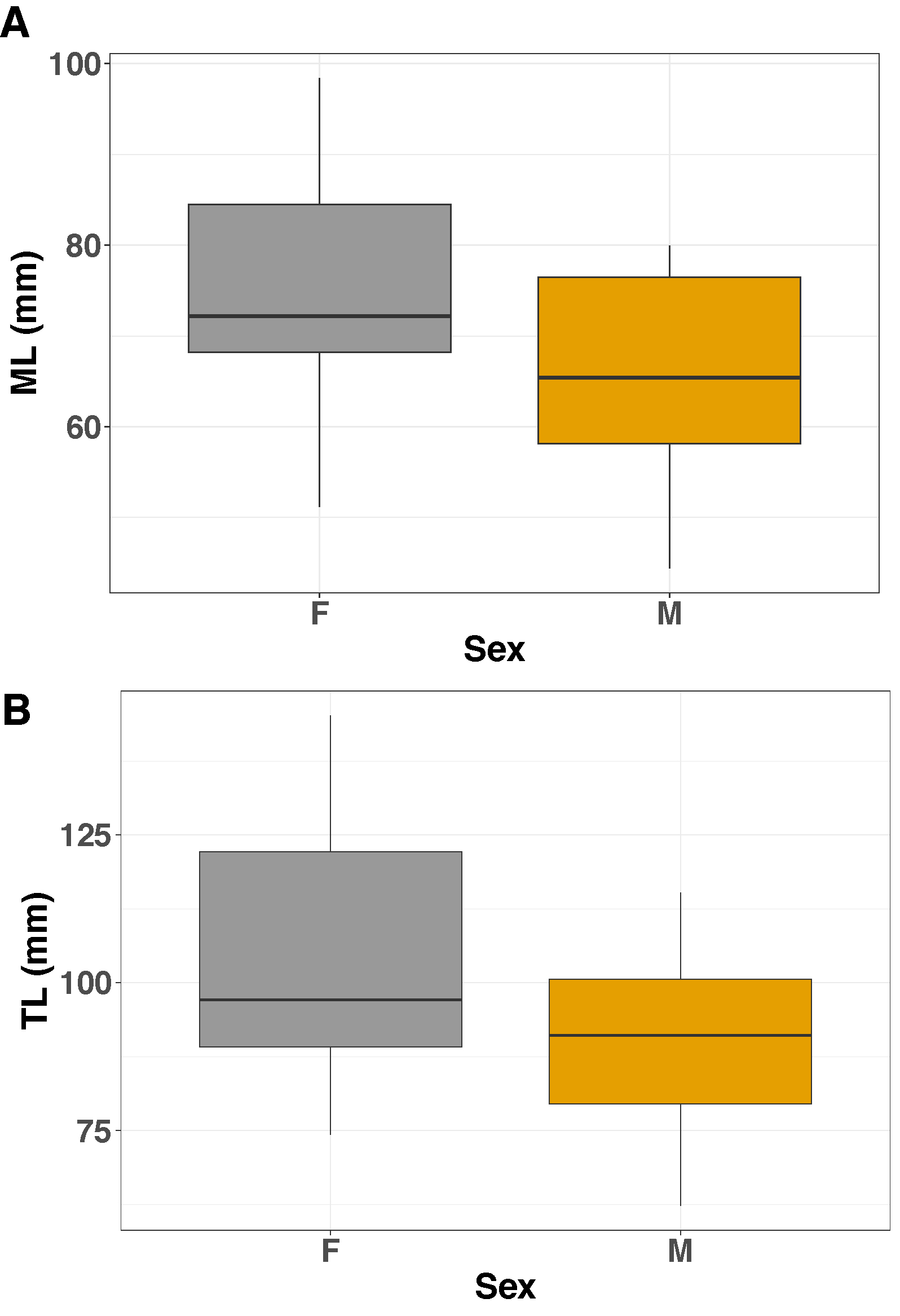

Supplement: Supplementary Figure 2 — (A) Mantle length (ML) and (B) Total length (TL) of mature females (gray boxplot, n = 34) and males (yellow boxplot, n = 32) S. plangon. [file Image_2.JPEG]
